# Discovery of thiazostatin D/E using UPLC-HR-MS2-based metabolomics and σ-factor engineering of *Actinoplanes* sp. SE50/110

**DOI:** 10.3389/fbioe.2024.1497138

**Published:** 2024-11-25

**Authors:** Laura Schlüter, Kine Østnes Hansen, Johan Isaksson, Jeanette Hammer Andersen, Espen Holst Hansen, Jörn Kalinowski, Yannik Karl-Heinz Schneider

**Affiliations:** ^1^ Microbial Genomics and Biotechnology, Center for Biotechnology, Bielefeld University, Bielefeld, Germany; ^2^ Department of Pharmacy, Faculty of Medicine and Health Sciences, UiT-The Arctic University of Norway, Tromsø, Norway; ^3^ Marbio, Faculty for Fisheries, Biosciences and Economy, UiT-The Arctic University of Norway, Tromsø, Norway; ^4^ Technology Platform Genomics, Center for Biotechnology, Bielefeld University, Bielefeld, Germany

**Keywords:** Actinobacteria, *Actinoplanes* sp. SE50/110, natural products, secondary metabolites, sigma factor, hydroxyphenylthiazoline, metabolomics

## Abstract

As the natural producer of acarbose, *Actinoplanes* sp. SE50/110 has high industrial relevance. Like most Actinobacteria, the strain carries several more putative biosynthetic gene clusters (BGCs) to produce further natural products, which are to be discovered. Applying a metabolomics-guided approach, we tentatively identified five further compounds that are produced by the strain: watasemycin, thiazostatin, isopyochelin, pulicatin, and aerugine. A comparison of the genomic context allowed the identification of the putative BGC, which is highly similar to the watasemycin biosynthetic gene cluster of *Streptomyces venezuelae*. In addition to the identified molecules, a thiazostatin-like compound was found. Isolation and structure elucidation with 1D and 2D NMR and HRMS were applied. The fraction containing *m/z* 369.0929 [M + H]^+^ comprised two highly similar compounds identified as thiazostatin D and thiazostatin E. The compounds possessed the same phenol–thiazole–thiazole molecular scaffold as the previously reported thiazostatin and watasemycin and have anti-proliferative activity against the breast adenocarcinoma cell line MCF7 and human melanoma cell line A2058, while no activity again the non-malignant immortalized fibroblast cell line MRC-5 was observed. We further showed that the manipulation of global transcriptional regulators, with *sigH* (*ACSP50_0507*) and anti-anti-σ factor coding *ACSP50_0284* as an example, enabled the production manipulation of the 2-hydroxyphenylthiazoline family molecules. While the manipulation of *sigH* enabled the shift in the peak intensities between the five products of this pathway, *ACSP50_0284* manipulation prevented their production. The production of a highly polar compound with *m/z* 462.1643 [M + H]^+^ and calculated elemental composition C_19_H_27_NO_12_ was activated under the *ACSP50_0284* expression and is exclusively produced by the engineered strain.

## 1 Introduction

The rise of antimicrobial resistance has become a global health crisis, with an increasing number of pathogens developing resistance to conventional antibiotics and causing many deaths (Murray et al., 2022). This leads to an increased demand for new and effective therapeutics (Rossiter et al., 2017; Schneider, 2021; Theuretzbacher et al., 2020). Natural products comprise diverse classes of bioactive molecules and are used as a source for therapeutics. In 2023, 79% of the antibiotics that were approved in the United States are natural products or their derivates (Walesch et al., 2023). In comparison to synthetic molecules, natural products have the advantage of a higher structural diversity and structural complexity than synthetic molecules (Atanasov et al., 2021). A sufficient source of natural products is microorganisms, being present in all environmental niches. Among bacteria, Actinobacteria are known for a wide variety of bioactive compounds with diverse activities such antibacterial, antifungal, antioxidant, anti-cancer, and anti-inflammatory properties (Almuhayawi et al., 2021; Azman et al., 2017; Jakubiec-Krzesniak et al., 2018; Salwan and Sharma, 2020). It is estimated that Actinobacteria still have large potential for the discovery of antimicrobial compounds (Watve et al., 2001).

Among Actinobacteria, members of the genus *Actinoplanes* are a rich source for natural product discovery. They produce a diverse range of bioactive compounds like antibacterial, antifungal, antitumoral, antioxidant, and antidiabetic activity. Well-known examples of antibiotics produced by members of *Actinoplanes* include lipiarmycin, ramoplanin, teichomycin, taitomycin, and teicoplanin (Coronelli et al., 1975; La Cruz et al., 2017; McCafferty et al., 2002; Parenti et al., 1978; Rudaia and Solov’eva, 1970; Somma et al., 1984). Further products have antifungal (Wagman et al., 1975; Yoo et al., 2017), antitumor (Breyer et al., 2011; Houchens et al., 1983; Xiang et al., 2010), and inhibitory activity against the HIV-1 integrase (Singh et al., 2002).

One important member of the genus is *Actinoplanes* sp. SE50/110, which is known as the natural producer of acarbose, an oligosaccharide-based α-glucosidase inhibitor used in the treatment of diabetes mellitus type 2. The filamentous and spore-forming Gram-positive, aerobic soil bacterium of the Actinomycetes family has, like other members of the phylum Actinobacteria, 71% G + C DNA content (Schwientek et al., 2011). The bacterium features a complex life cycle and morphological differentiation. Like other members of the genus *Actinoplanes*, it shows mycelium growth, forming hyphae and motile spores (Parenti and Coronelli, 1979). In addition to acarbose, *Actinoplanes* sp. SE50/110 is the producer of an unidentified carotenoid (Droste et al., 2020), the pigment eumelanin (Wolf et al., 2016), and an unidentified pyochelin-related molecule (Wendler et al., 2015). The production of further metabolites is suspected (Wolf et al., 2017).

Natural product discovery is challenged by the broad inactivity of biosynthetic gene clusters (BGCs) under standard laboratory conditions since they are not essential for primary growth and survival (Liu J. et al., 2021). The production increase or activation of silent gene clusters can be facilitated by diverse variations of cultivation conditions (Akhter et al., 2018; Dashti et al., 2017; Mo et al., 2013; Sharma et al., 2017) and co-cultivation (Abdel-Razek et al., 2018; El-Hawary et al., 2018; Wakefield et al., 2017; Yu et al., 2019). Molecular genetic approaches like recombinant cluster expression and engineering of biosynthetic gene clusters can be applied (Izawa et al., 2013; Waldman et al., 2015; Yamanaka et al., 2014), especially when optimization of the cultivation conditions is not sufficient. Natural product synthesis can also be activated or enhanced by the manipulation of transcriptional regulators (Bunet et al., 2011; Laureti et al., 2011; Lin et al., 2006; Sidda et al., 2014).

Since σ factors are one of the largest groups of transcriptional regulators, their manipulation offers great potential to manipulate complex cellular processes. They confer promoter recognition to the RNA-polymerase core enzyme and thereby enable transcription (Browning and Busby, 2016; Tripathi et al., 2014), whereas housekeeping σ factors enable essential cellular processes, and alternative σ factors enable the transcription of alternative σ-factor regulons for specific processes like cell cycle, development, stress responses, biofilm formation, and natural product formation (Paget, 2015). Thereby, σ factors are involved in complex regulatory networks. Different regulations of σ factors are known, one of which is the partner switching mechanism, where a σ factor can be inhibited by its anti-σ factor, which itself can be inhibited by its cognate anti-anti-σ factor. Previously, σ factors were shown to pose as a sufficient target for the manipulation of gene expression profiles in Actinobacteria to increase secondary metabolite production (Jiang et al., 2011; Kumar et al., 2015; Schlüter et al., 2024; Seipke et al., 2014; Taniguchi et al., 2017).

In this study, we investigated the potential of *Actinoplanes* sp. SE50/110 for the discovery of natural products and unveiled six unidentified metabolites in *Actinoplanes* sp. SE50/110. Regarding the genomic potential of *Actinoplanes* sp. SE50/110 for the synthesis of natural products, we applied σ-factor engineering. We demonstrate that σ-factor and anti-anti-σ-factor engineering can be applied to activate a silent gene cluster and render the product distribution within a biosynthesis pathway in *Actinoplanes* sp. SE50/110.

## 2 Materials and methods

### 2.1 Strains used in this study

For the creation of sigma-factor mutant strains, *Escherichia coli DH5αMCR* (Grant et al., 1990) was used as the cloning host, *E. coli ET12567* (pUZ8002) was used as the donor strain for conjugation into *Actinoplanes* sp. SE50/110, and *E. coli* ER2925 was used as the methylase-free plasmid replicant strain for transformation into *Actinoplanes* sp. SE50/110.

### 2.2 Cultivation

*Actinoplanes* strains were grown on a solid soy flour medium (SFM: 20 g L^−1^ soy flour, 20 g L^−1^ mannitol, and 20 g L^−1^ agar, pH 8 adjusted with NaOH and tap water). For the pre-culture, 30 mL NBS (11 g ·L^−1^ glucose × 1H_2_O, 4 g ·L^−1^ peptone, 4 g ·L^−1^ yeast extract, 1 g ·L^−1^ MgSO_4_·7H_2_O, 2 g ·L^−1^ KH_2_PO_4_, and 4 g ·L^−1^ K_2_HPO_4_) was inoculated in a plate and cultivated for 72 h at 28°C and 140 rpm in shake flasks. Cultivation for dereplication was performed using 350-mL baffled cell culture flasks for 70 mL (wild-type) or 50 mL (mutants) culture at 28°C and 140 rpm in a maltose minimal medium [(1) 72.06 g ·L^−1^ maltose**·**1H_2_O, (2) 5 g ·L^−1^ (NH_4_)2SO_4_, 0.184 g ·L^−1^ FeCl_3_·4H_2_O, 5.7 g ·L^−1^ Na_3_C_6_H_5_O_7_·2H_2_O, 1 g ·L^−1^, 200 µL trace elements (stock concentration: 1 μM CuCl_2_, 50 μM ZnCl_2_, and 7.5 μM MnCl_2_), (3) MgCl_2_.6H_2_O, 2 g ·L^−1^ CaCl_2_·2H_2_O, and (4) 5 g ·L^−1^ each K _2_HPO_4_ and KH_2_PO_4_]. Inoculation was performed using 1 mL pre-culture, which was harvested by centrifugation at 5,000 rpm for 5 min and washed twice with the maltose minimal medium. Cells were dissolved in 1 mL maltose minimal medium, of which 200 µL was used to inoculate 80 mL maltose minimal medium. The cultivation was performed for 7 days. For the extraction of compounds of interest, 5.5 L bacterial cell culture was cultivated in 250-mL baffled cell culture flasks (Corning Inc., Corning, NY, United States) at 28°C and 140 rpm for 7 days.

### 2.3 Extraction

General procedures: pH_2_O was produced using the in-house Milli-Q system.

For UPLC-MS2 analysis and dereplication, 70 mL (wild-type) and 50 mL (mutants) cultivation broths were frozen in separate glass beakers at −80°C for 20 min, before freeze-drying for 2 days. The dried cultures were then wetted with pH_2_O and extracted with 70 mL MeOH (*v/v*; HiPerSolv, VWR, Radnor, Penns., United States). After stirring for 4 h using a magnetic stirrer, the extraction broth was vacuum-filtered using a Büchner funnel and filter paper (Whatman No. 3, 6 µm pore size). To exclude medium components in the analysis, the extraction process was also performed for the medium. The extracts were then stored at −20°C until dry under pressure and 40°C.

For isolation of compounds, *Actinoplanes* sp. SE50/110 was cultivated in 5.5 L maltose minimal medium for 7 days with 50 mL in each 250-mL baffled cell culture flasks (Corning Inc., Corning, NY, United States) and frozen at −20°C until extraction. Therefore, Diaion HP-20 resin (Supelco, Bellefonte, PA, United States) was activated by incubation with methanol for 30 min and subsequent washing with pH_2_O for 15 min, before the addition of 60 g·L^−1^ activated resin to 1.4 L defrosted bacterial culture and further incubated for 4 days at room temperature and under movement. The beads were harvested by vacuum filtration through a cheesecloth mesh (X001LCT5PF, Dueco, Wolfsburg, Germany) using a Büchner funnel and washed with 150 mL pH_2_O. The molecules extracted from the resin were eluted two times in 200 mL 100% methanol for 30 min under shaking. Vacuum filtration was used to separate the resin from the methanol and a final filtration through a filter paper (Whatman No. 3, 6 µm pore size, Sigma-Aldrich). The crude extract was dried under pressure at 40°C, and 11.558 g of the dried extract was received and stored at −20°C until further use.

### 2.4 Fractioning using FLASH chromatography

For fractionation of the extract, FLASH chromatography (Biotage SP4TM system, Uppsala, SE) was performed using a water–methanol–acetone step gradient with Diaion HP20-SS resin (Supelco, Bellefonte, PA, United States) as the stationary phase for the wild-type extract, pSETT4-0284 extract, and the media control. Then, 1.5 g extract was re-dissolved in 10 mL 90% MeOH (*v/v*), and 1.5 g resin was added. The mixture was dried under pressure at 40°C before adding to the column of pre-loaded 4 g activated resin in 5% MeOH in a flash cartridge (Biotage, Uppsala, Sweden). The extract was separated into 15 fractions under a pH_2_O:MeOH gradient of 5%–100% MeOH (*v/v*; HiPerSolv, VWR, Radnor, Penns., United States) over 32 min with a flow rate of 12 mL ·min^−1^ and in further three fractions with an MeOH:acetone gradient to 100% acetone for 18 min and a flow rate of 12 mL ·min^−1^. The eight collected fractions were then dried under pressure at 45°C (Buchi Syncore^®^ Polyvap, Flawil, Switzerland), afterward solved in DMSO at 10–80 mg ·mL^−1^, and stored at −20°C until further use.

### 2.5 UHPLC-MS/MS analysis and de-replication

The extracts were diluted to 1:3 (*v/v*) with 80% MeOH (*v/v*) for analysis using UHPLC-QToF-MS. The UHPLC system (Acquity I-class, Waters, Milford, MA, United States) was coupled to a PDA detector (Waters, Milford, MA, United States) and Vion IMS QToF (Waters, Milford, MA, United States). The Acquity UPLC BEH C18 column (1.7 µm, 2.1 × 1.5 mm) (Waters, Milford, MA, United States) was used for the separation over an 10%–100% gradient of acetonitrile (HiPerSolv, VWR, Radnor, Penns., United States) with pH_2_O, both supplemented with 0.1% formic acid (*v/v*; 33015; Sigma-Aldrich), in 12 min with a flow rate of 0.2 mL ·min^−1^. A 5-µL sample was injected at an infusion rate of 15 μL ·min^−1^, and mass spectrometry was run with a column temperature of 40°C with positive electrospray ionization. Leucine-enkephalin (100 pg ·μL^−1^) was used as an internal standard. As controls, the medium fractions and MeOH were analyzed as well.

### 2.6 Isolation of the compound using preparative HPLC and mass-triggered fractioning

Preparative reversed-phase HPLC was used for the isolation of compounds from flash fractions. The collection of the respective fractions for the first and second rounds of purification was triggered by the recorded mass signal throughout the chromatographic separation. The HPLC system used consisted of a Waters 600 HPLC -pump with an additional degasser and flow-splitter (all Waters), a Waters 515 HPLC pump functioning as a “make-up” pump, a Waters 2996 photoarray detector, a Waters 3100 Mass detector, and a Waters 2767 sample manager. For system control, MassLynx V4.1 (Waters) software was used. As stationary phases, a SunFire RP-18 preparative column (10 μm, 10 mm × 250 mm) and XSelect CSH preparative Fluoro-Phenyl column (5 μm, 10 mm × 250 mm) (both Waters) were used. As mobile phases A [pH_2_O with 0.1% (*v/v*) formic acid] and B [acetonitrile with 0.1% (*v/v*) formic acid], different gradients at a flow rate of 6 mL·min^−1^ were used for separation. Acetonitrile (Prepsolv^®^, Merck KGaA, Darmstadt, Germany) and formic acid (33015, Sigma-Aldrich) were purchased in appropriate quality, and ddH_2_O was produced using the in-house Milli-Q^®^ system. For the mass-triggered fractionation, 1% of the eluent was split and blended with 80% MeOH in pH_2_O (*v/v*) acidified with 0.2% formic acid (Sigma-Aldrich) and directed to the ESI-quadrupole-MS. The fractions were collected by mass-triggered fraction collection controlled by MassLynx, and the respective fractions were reduced to dryness under reduced pressure and by vacuum centrifugation, both at 40°C.

### 2.7 Bioactivity screening

#### 2.7.1 Anti-microbial assay

The compounds were tested for potential anti-microbial activity against S*taphylococcus aureus* (ATCC 25923), *E. coli* (ATCC 259233), and S*treptococcus agalactiae* (ATCC 12386). All isolates were provided by LGC Standards (Teddington, London, United Kingdom). *S. aureus* and *E. coli* were grown in Muller–Hinton broth (275730, Becton). *S. agalactiae* was cultured in brain heart infusion broth (53286, Sigma-Aldrich). Fresh bacterial colonies were transferred into the respective medium and incubated at 37°C overnight. The bacterial cultures were diluted to a culture density representing the log phase, and 50 μL/well was pipetted into a 96-well microtiter plate (734–2097, Nunclon™, Thermo Scientific, Waltham, MA, United States). The final cell density was 1,500–15,000 colony-forming units/well. Extracts were diluted in 2% (*v/v*) dimethyl sulfoxide (DMSO) in pH_2_O, and the final assay concentration was 50% of the prepared sample as 50 μL of the sample in DMSO/water was added to 50 μL of the bacterial culture. After adding the samples to the plates, they were incubated overnight at 37°C, and the growth was determined by measuring the optical density at λ = 600 nm (OD_600_) using a VICTOR3™ 1420 Multilabel Counter (PerkinElmer, Waltham, MA, United States). A water sample was used as the reference control, a growth medium without bacteria as a negative control, and a dilution series of gentamicin (32–0.01 μg ·mL^−1^, A2712, Merck) as the positive control and visually inspected for bacterial growth. The positive control was used as a system suitability test, and the results of the antimicrobial assay were only considered valid when the positive control was passed.

#### 2.7.2 Anti-proliferative assay

The compound(s) were investigated for potential anti-proliferative activity against human cell lines by an anti-proliferative assay using death–life staining. The cell lines tested were MRC-5 (lung fibroblasts, ATCC CCL-171™), MCF7 (human breast carcinoma, ATCC HTB-22), A2058 (human melanoma, ATCC CRL-11147), Molm13 (acute myeloid leukemia, ATCC CRL-2003), and MDA-MB-231 (mammary gland, epithelial adenocarcinoma, ATCC HTB-26). The cells were cultured and assayed in Roswell Park Memorial Institute medium (RPMI-16040, FG1383, Merck) containing 10% (*v/v*) fetal bovine serum (FBS, 50115, Biochrom, Holliston, MA, United States). The cell concentration was 2 × 10^4^ cells for the Molm13 cell line and 15 × 10^3^ cells for the other cell lines. After seeding, the cells were incubated for 24 h at 37°C and 5% CO_2_. The medium was then replaced with fresh RPMI-1640 medium supplemented with 10% (*v/v*) FBS and gentamicin (10 μg·mL^−1^, A2712, Merck). After adding 10 μL of extract samples diluted in 2% (*v/v*) DMSO in pH_2_O, the cells were incubated for 72 h at 37°C and 5% CO_2_. For assaying the viability of the cells, 10 μL of CellTiter 96 Solution reagent (G3581, Promega, Madison, WI, United States) containing tetrazolium ^®^ AQueous One [3-(4,5-dimethylthiazol-2-yl)-5-(3carboxymethoxyphenyl)-2-(4-sulfophenyl)-2H-tetrazolium, inner salt] and phenazine ethosulfate were added to each well and incubated for 1 h. The assays were done with three technical replicates. The plates were read using a DTX 880 plate reader (Beckman Coulter, CA, United States) by measuring the absorbance at λ = 485 nm. The cell viability was calculated using the media control. As a negative control, RPMI-1640 with 10% (*v/v*) FBS and 10% (*v/v*) DMSO (Sigma) was used as a positive control.

#### 2.7.3 Siderophore activity

The supernatant of the wild-type and mutant strains was tested for siderophore activity using the chrome azurol S (CAS) assay. Therefore, 25 mL 2.4 mM hexadecyl trimethyl ammonium bromide (HDTMA) solution was slowly mixed with 1.5 mL CAS solution (10 mM HCl, 1.7 mM FeCl_3_, 7.5 mL CAS solution (2 mM Chrome Azurol S in H_2_O)), before 50 mL of 0.915 M 2-morpholinoethanesulfonic acid monohydrate (MES) was added. The assay was performed with 100 µL assay solution and 100 µL supernatant in 96-well microtiter plates. The mixture was incubated in the dark and at room temperature for 1 h before absorption measurement at 630 nm using the Tecan Infinite M200 microplate reader and i-control 10.1 software (Tecan Group AG, Switzerland).

### 2.8 NMR analysis

The structures of thiazostatin D/E were elucidated by 1D and 2D NMR spectroscopy. NMR spectra were acquired in DMSO-d6 at 298 K, in 3-mm solvent-matched Shigemi tubes, on a Bruker Avance III HD spectrometer (Bruker, Billerica, MA, United States) at 600 MHz, equipped with an inverse TCI cryo-probe enhanced for 1H, 13C, and 2H. Spectroscopy was performed under standard pulse programs for proton, carbon, HSQC, HMBC, COSY, and ROESY.

### 2.9 Recombinant DNA work

#### 2.9.1 Cloning of the *sigH* and *ACSP50_0284* expression plasmids

The additional copy of different sigma factor-related genes was cloned into the BbsI-linearized vector pSETT4*tipA* using Gibson Assembly (Gibson et al., 2009) according to Schaffert et al. (2019). Used oligonucleotides were ordered from metabion GmbH (Steinkirchen, Germany). Target genes were amplified with the according oligonucleotides (Supplementary Table S1) by polymerase chain reaction (PCR) using Phusion Flash High-Fidelity PCR Master Mix (Thermo Fisher Scientific, Waltham, MA, United States). DNA constructs were transformed into *E. coli* DH5α according to Beyer et al. (2015) and selected on solid Luria/Miller broth (LB media) (Carl Roth, GmbH&Co. KG, Karlsruhe, Germany) with 16 g ·L^−1^ agar-agar KobeI (Carl Roth, GmbH&Co. KG, Karlsruhe, Germany), supplemented with 50 mg ·L^−1^ apramycin sulfate. Plates were incubated overnight at 37°C, and obtained clones were screened using colony PCR and agarose gel electrophoresis. Positive clones were isolated using the GeneJET Plasmid Miniprep Kit (Thermo Fisher Scientific, Waltham, MA, United States) and verified by Sanger sequencing by our in-house sequencing core facility.

#### 2.9.2 Cloning of the CRISPR/Cas9-based *sigH* and *ACSP50_0284* deletion plasmids

Deletion mutants were created using the CRISPR/Cas9-based vector pCRISPomyces-2 (Cobb et al., 2015). The protospacer sequences were selected based on the CRISPy-web tool (Blin et al., 2016) and ordered without 3′ PAM sequence as the oligonucleotide, as well as its reverse complement, both including a 4-bp overlap to the integration site of the vector (Metabion GmbH, Steinkirchen, Germany). Oligonucleotide annealing of 5 μL, 10 mM oligonucleotides (Supplementary Table S1) was performed in 30 mM HEPES of pH 7.8 for 1 h at RT and cloned into the BsaI (NEB, Ipswich, MA, United States) linearized vector by Golden Gate Assembly according to Cobb et al. (2015). The DNA constructs were transformed into chemocompetent *E. coli* DH5α according to Beyer et al. (2015) and selected on solid LB media (Carl Roth, GmbH&Co. KG, Karlsruhe, Germany), supplemented with 16 g L^−1^ agar-agar KobeI (Carl Roth, GmbH&Co. KG, Karlsruhe, Germany), 10 µL 0.8 M IPTG, 40 µL xGal, and 50 mg L^−1^ apramycin sulfate. Plates were incubated overnight at 37°C, and blue–white screening was used to screen positive clones, which then were isolated using the GeneJET Plasmid Miniprep Kit (Thermo Fisher Scientific, Waltham, MA, United States).

Homologous flanks of ∼1.3 kb, operating as templates for the repair of the Cas9-induced double-strand break, were amplified by PCR using Phusion Flash High-Fidelity PCR Master Mix (Thermo Fisher Scientific, Waltham, MA, United States). The homologous flanking regions were amplified and cloned in the XbaI linearized vector–spacer construct by Gibson Assembly (Gibson et al., 2009). Transformation was performed as mentioned previously, and colony PCR and agarose gel-electrophoresis were applied for screening. Positive clones were isolated using the GeneJET Plasmid Miniprep Kit (Thermo Fisher Scientific, Waltham, MA, United States) and verified by Sanger sequencing by our in-house sequencing core facility.

#### 2.9.3 Plasmid transfer into *Actinoplanes* sp. SE50/110

Competent cells were obtained from 50 mL NBS culture and cultivated for 48 h at 140 rpm and 28°C. After 15 min on ice, the cells were washed two times with TG buffer (10% glycerol (*v/v*); 1 mM Tris) and once in 10% (*v/v*) glycerin with a centrifugation of 5 min at 5,000 g and 4°C in between. The cells are then resuspended in 3 mL of 10% (*v/v*) glycerol, and aliquots of 300 µL were prepared and subsequently frozen in liquid nitrogen and stored at −80°C until further use. Electro-transformation of the pSETT4*tipA*-based expression plasmids into competent *Actinoplanes* sp. SE50/110 was performed with 1 µL of the overexpression plasmid, isolated from the methylase-deprived *E. coli* ER2925 strain, at 2,500 kV, 25 μF, 200 Ω in a cuvette and incubated in 1 mL preheated CMR (10 g ·L^−1^ glucose, 103 g L^−1^ sucrose, 10.12 g ·L^−1^ MgCl_2_.6H_2_O, 15 g L^−1^ TSB, and 5 g L^−1^ yeast extract) at 46°C for 6 min before incubation at 28°C and 1,100 rpm overnight. The transformation broth was then grown on solid selective solid SFM agar, supplemented with 50 mg L^−1^ apramycin sulfate, and clones were screened by colony PCR and agarose gel-electrophoresis. Genomic integration was verified by Sanger Sequencing and Oxford Nanopore sequencing (Supplementary Figure S1, S3) using the MinION (Oxford, United Kingdom) after isolation from NBS culture using the Quick-DNA Fungal/Bacterial Miniprep (Zymo Research Corporation, Irvine, CA, United States).

The deletion plasmids were conjugated with competent *Actinoplanes* sp. SE50/110 cells and *E. coli* ET12567/pUZ8002 as the donor according to the protocol in the study by Schaffert et al. (2019). Plasmid curing of exconjugants was performed according to Wolf et al. (2016), and the deletion was tested by PCR. Genome sequencing after gDNA isolation of NBS culture using Quick-DNA Fungal/Bacterial Miniprep (Zymo Research Corporation, Irvine, CA, United States) was performed using ONT MinION by Oxford Nanopore (Oxford, United Kingdom) (Supplementary Figures S2, S4).

### 2.10 Software and databases used for bioinformatics analysis

Genetic engineering was planned, and oligonucleotides were designed using SnapGene 4.3 (GSL Biotech LLC, Chicago, IL, United States). AntiSMASH 7.0 (Blin et al., 2023) was used for comparative genome studies of secondary metabolite clusters, and UNIFI 1.9.4 Scientific Information System Software (Waters) was used for data processing and computational prediction of elemental composition. PubChem and ChemSpider were used for compound identification. Compound masses and isotopic distributions were calculated using ChemCalc (Patiny and Borel, 2013), and Collision Cross Section was determined using CCSbase (Ross et al., 2020). Visualization was created using BioRender.com.

## 3 Results

### 3.1 Genomic potential of *Actinoplanes* sp. SE50/110 for metabolite biosynthesis

Since Actinobacteria are known for their large genomic potential for natural product biosynthesis, the *Actinoplanes* sp. SE50/110 genome was investigated for putative secondary metabolite biosynthesis gene clusters. This offers the advantage to expand our knowledge not only on strongly produced metabolites but also on low expressed or silent secondary metabolite gene clusters of already established strains. Prior analysis showed 20 suspected BGCs of *Actinoplanes* sp. SE50/110, of which 3 metabolites were previously known: acarbose, eumelanin, and an unidentified carotenoid (Wolf et al., 2017). Recent analysis using antiSMASH 7.0 identified 19 putative secondary metabolite biosynthetic gene clusters.

The recent analysis also added redefined product classes, with NAPAA (non-alpha poly-amino acids like ε-Poly-L-lysine), LAP (linear azol (in)e-containing peptides), RRE-containing, and RiPP-like clusters. Two putative metabolite gene clusters were identified with a similarity of 86% and 100% to biosynthetic clusters of known metabolites. These clusters contain genes for the biosynthesis of NRPS, T1PKS, NRP-metallophore-type, and NAPPA-type molecules, similar to the watasemycin biosynthesis of *Streptomyces venezuelae* and the ε-Poly-L-lysine biosynthesis of *Epichloe festucae*. A low similarity of <80% was determined for most coded putative gene clusters to known metabolite clusters (Table 1), which underlines the potential for the identification of novel molecules *Actinoplanes* sp. SE50/110.

**TABLE 1:** *Actinoplanes* sp. SE50/110 metabolite gene clusters. Analysis was performed using antiSMASH7.0 and MIBiG.

Gene cluster	Gene cluster type	Location [bp]	Known cluster with highest similarity	Cluster similarity [%]	MIBiG accession Id (BGC)	Species	References
1	Terpene	141,289–162,176	Isorenieratene	25	0,001,456	*Streptomyces argillaceus*	Becerril et al. (2018)
2	NRPS, lassopeptide, and betalactone	994,953–1,075,026	Salinamide A/B/C/D/E/F desmethylsalinamide	14	0,001,230	*Streptomyces* sp. *CNB091*	Trischman et al. (1994)
3	Terpene	2,149,358–2,170,386	Legonindolizidine A6	12	0,002,666	*Streptomyces* sp. *MA37*	Wang et al. (2022)
4	NRPS	3,373,041–3,429,926	Bosamycin A/B/C/D/E/F	44	0,002,581	*Streptomyces* sp.	Xu et al. (2020)
5	LAP	3,483,661–3,507,985	α-Lipomycin	9	0,001,003	*Streptomyces aureofaciens*	Bihlmaier et al. (2006)
6	NAPAA	3,655,404–3,689,309	ε-Poly-L-lysine	100	0,002,174	*Epichloe festucae*	Purev et al. (2020)
7	RiPP-like	3,960,208–3,972,124	-	-	-	*-*	-
8	amglyccycl	4,067,209–4,088,405	Acarbose	57	0,000,691	*Actinoplanes* sp. *SE50/110*	Schwientek et al. (2012)
9	Thiopeptide	4,153,425–4,185,196	Rapamycin/globomycin	72	0,001,753	*Streptomyces globisporus*	
10	NRPS, lanthipep-tide-class-ii	4,504,073–4,571,975	Vacidobactin A/B	11	0,002,420	*Variovorax paradoxus S110*	Johnston et al. (2015)
11	NI-siderophore	4,772,033–4,803,040	-	-	-	*-*	-
12	Lanthipeptide-class-iv	4,934,219–4,956,963	Labyrinthopeptin A1/A2/A3	40	0,000,519	*Actinomadura namibiensis*	Meindl et al. (2010)
13	Melanin	5,449,982–5,462,241	-	-	-	*-*	-
14	T1PKS and ranthipeptide	6,550,938–6,678,259	Salinilactam	56	0,000,142	*Salinispora tropica CNB-440*	Udwary et al. (2007)
15	NRPS, T1PKS, and NRP-metallophore	6,707,893–6,793,765	Thiazostatin/watase-mycin/2-hydroxy-phenylthiazoline enantiopyochelin/isopyochelin	86	0,001,801	*Streptomyces venezuelae ATCC 10712*	Inahashi et al. (2017)
16	NRPS-like, NRPS	6,794,964–6,841,369	Cadaside A/B	14	0,001,968	Uncultured bacterium	Wu et al. (2019)
17	RRE-containing	6,854,877–6,875,161	-	-	-	*-*	-
18	NI-siderophore, T1PKS	7,710,720–7,782,831	Desferrioxamine E	75	0,001,572	*Pantoea agglomerans*	Smith and Strobel (2011)
19	T3PKS	8,838,789–8,879,853	Loseolamycin A1/A2	60	0,002,362	*Micromonospora endolithica*	Lasch et al. (2020)

### 3.2 Dereplication reveals five products of the 2-hydroxyphenylthiazoline family

**FIGURE 1:**
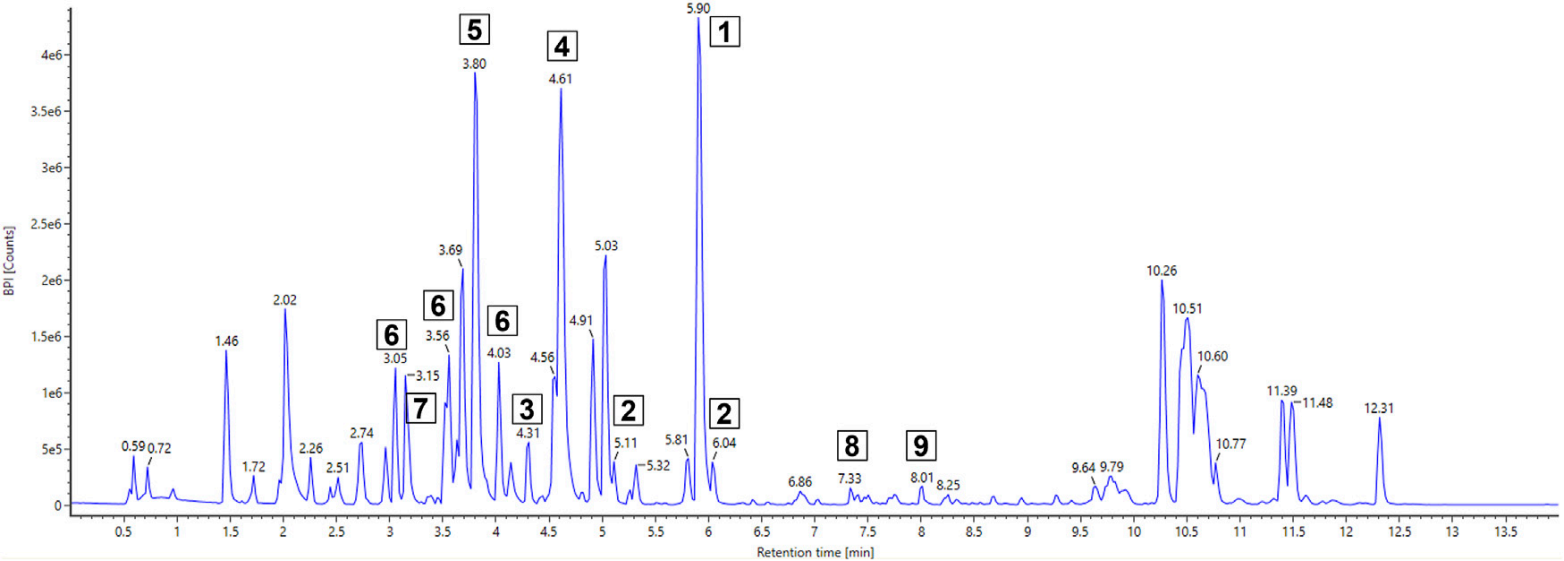
BPI chromatogram of FLASH fraction 5 of *Actinoplanes* sp. SE50/110 whole-cell extract using UHPLC-QToF-MS. The fraction was dissolved in DMSO to 20 mg·mL^−1^and diluted 1:3 (*v/v*) to 6.67 mg·mL^−1^with 5 µL injected. Compounds of interest are numbered.

For the analysis of *Actinoplanes* sp. SE50/110 MeOH whole-cell extract, the focus was set on dominant peaks indicating a production in sufficient amounts for further compound isolation and structure elucidation. It also sets the focus on compounds which are produced in resource competition to the acarbose biosynthesis, and further knowledge about the metabolites can be beneficial for future strain engineering approaches. Several dominant compounds were found, and their elemental composition was computationally calculated using UNIFI 1.9.4 (Figure 1). It revealed seven sulfur-containing compounds (Table 2). These compounds were mostly present in fraction 5 but were also found in fractions 6 and 7 and partly in fractions 4 and 8. A molecular mass difference of 14 kDa and similar elemental compositions of compounds 1–3 (1: *m/z* 353.0988 [M + H]^+^ with C_16_H_20_N_2_O_3_S_2_; 2: *m/z* 339.0836 [M + H]^+^ with C_15_H_18_N_2_O_3_S_2_; 3: *m/z* 325.0656 [M + H]^+^ with C_14_H_16_N_2_O_3_S_2_), compounds 4 and 5 (4: *m/z* 224.0743 [M + H]^+^ with C_11_H_13_N O_2_S and 5: *m/z* 210.0583 [M + H]^+^ with C_10_H_11_N O_2_S), and compounds 6 and 7 (6: *m/z* 369.0929 [M + H]^+^ with C_16_H_20_N_2_O_4_S_2_ and 7: *m/z* 355.0779 [M + H]^+^ with C_15_H_18_N_2_O_4_S_2_) leads to the assumption of a putative biosynthetic connection of compounds 1–7.

**TABLE 2:** Compounds of interest of *Actinoplanes* sp. SE50/110 fraction 5 whole-cell extract. The elemental composition and charge were predicted using UNIFI 1.9.4 (Waters). The mass-to-charge-ratio and their fragments of the high energy spectrum with the respective predicted elemental compositions are shown for all compounds.

Compound number	Observed retention times [min]	Charge	*m/z* *(Fragments)*	Elemental composition *(Fragments)*	Putative similar product
1	5.04 5.92	[M + H]^+^	353.0988 *160.0426* *204.0477* *200.0738*	C_16_H_20_N_2_O_3_S_2_ *C_6_H_9_ N O_2_S* *C_11_H_9_ N O S_2_* *C_9_ H_13_N O_2_S*	Watasemycin A/B^a^
2	4.44 5.11 5.32 6.04 6.62	[M + H]^+^	339.0836 *204.0482*	C_15_H_18_N_2_O_3_S_2_ *C_11_H_9_N O S*	Thiazostatin A/B^a^
3	4.01 4.31	[M + H]^+^	325.0656 *190.0321* *172.0428* *151.3725*	C_14_H_16_N_2_O_3_S_2_ *C_10_H_7_N OS* *C_7_H_9_N O_2_S* *NA*	(Iso)Pyochelin^a^
4	4.56 4.61	[M + H]^+^	224.0743 *178.0319*	C_11_H_13_N O_2_S *C_9_H_7_N O S*	Pulicatin A/B^a^
5	3.80	[M + H]^+^	210.0583 *178.0319*	C_10_H_11_N O_2_S *C_9_H_7_N O S*	Aerugine^a^
6	3.05 3.56 4.03 4.59	[M + H]^+^	369.0929 *220.0425* *160.0425*	C_16_H_20_N_2_O_4_S_2_ *C_11_H_9_NO_2_S* *C_6_H_9_NO_2_S*	-
7	3.15 3.52 3.76 4.23 4.82	[M + H]^+^	355.0779 *160.0427*	C_15_H_18_N_2_O_4_S_2_ *C_6_H_9_NO_2_S*	-
8	7.33 7.41 7.58 7.63	[M + H]^+^	440.2768 *299.2580*	C_23_H_33_N_7_O_2_ *NA*	-
9	8.01 8.14 8.25 8.39	[M + H]^+^	454.2917 *313.2735*	C_24_H_35_N_7_O_2_ *NA*	-

^a^ Similar product from *Streptomyces venezuelae*.

Further detected promising compounds are compounds 8 and 9 with *m/z* 440.2768 [M + H]^+^ and *m/z* 454.2917 [M + H]^+^, for which the elemental compositions C_23_H_33_N_7_O_2_ and C_24_H_35_N_7_O_2_ were predicted. Despite comprehensive literature and database research, no similar molecules could be determined for both compounds, which are suspected as putative novel molecules. Unfortunately, their low-intensity peaks present a challenge, indicating limited amounts in the already concentrated fractions and even less in the cell extract. This poses a critical challenge for its isolation and further analysis as substantial quantities are required for structure elucidation. That is why no isolation and identification attempts were made.

**FIGURE 2:**
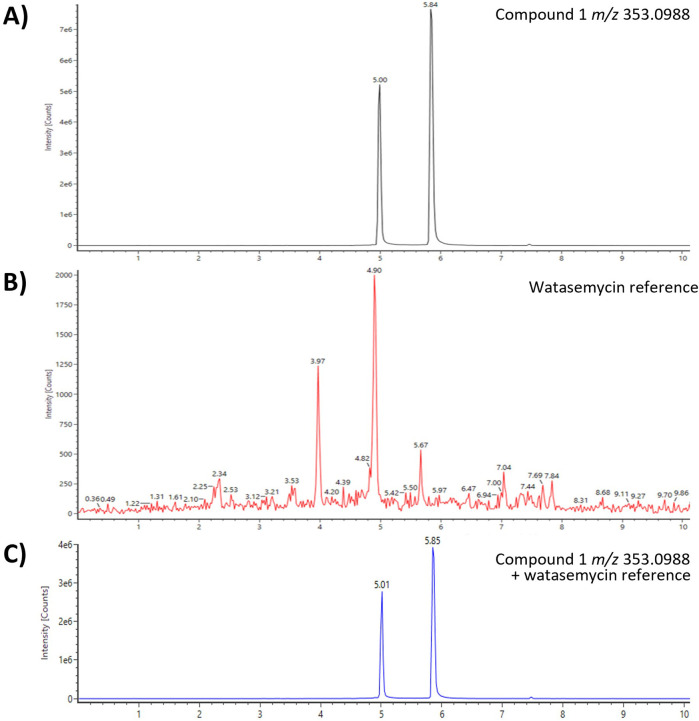
UHPLC-QToF-MS of *m/z* 353.0988 and a watasemycin reference identified compound 1 as watasemycin (retention time of 4.90–5.01 min). **(A)** Compound 1 of *Actinoplanes* sp. SE50/110 fraction 5, **(B)** watasemycin reference, and **(C)** co-injection of fraction 5 and reference sample.

Comprehensive literature and database research of the elemental composition and fragmentation pattern of compound 1 showed that it corresponds with watasemycin. Watasemycin (C_16_H_20_N_2_O_3_S_2_) is a natural product of *Streptomyces* sp. TP-A0597*,* of which two isoforms are known so far (Sasaki et al., 2002). It is a natural product of *S. venezuelae,* where the watasemycin biosynthesis pathway has four further products: thiazostatin (C_15_H_18_N_2_O_3_S_2_), isopyochelin (C_14_H_16_N_2_O_3_S_2_), pulicatin (C_10_H_11_NO_2_S), and aerugine (C_10_H_11_NO_2_S) (Inahashi et al., 2017). Thiazostatin has antioxidant activity (Shindo et al., 1989), pyochelin and pulicatin are siderophores with iron-chelating activity (Cox et al., 1981; Lin et al., 2010), and aerugine has antifungal and anti-oomycete activity (Lee et al., 2003). Interestingly, these known products have an identical elemental composition to the predicted composition of compounds 2–5. Since the genomic analysis determined a cluster similarity of 86% of cluster 15 and the *S. venezuelae* watasemycin biosynthesis gene cluster, compounds 1–5 are suspected as watasemycin, thiazostatin, isopyochelin, pulicatin, and aerugine of a putative similar watasemycin biosynthesis pathway in *Actinoplanes* sp. SE50/110. Analysis of a watasemycin reference enabled the confirmation of this assumption. When compared to the extract fraction dataset, the synthetic watasemycin eluted at a slightly earlier retention time but the extracted mass chromatogram of an analyzed mixed sample confirmed that compound 1 is watasemycin (Figure 2). Consequently, compound 2 is assigned as thiazostatin, compound 3 as isopyochelin, compound 4 as pulicatin, and compound 5 as aerugine. This adds five further identified products to two already known products of *Actinoplanes* sp. SE50/110.

### 3.3 Comparative genomics reveals an extended watasemycin gene cluster

The biosynthesis of watasemycin, thiazostatin, isopyochelin, pulicatin, and aerugine was investigated in *S. venezuelae* in 2017 (Inahashi et al., 2017). Nine genes encode biosynthesis proteins, which are essential for the formation of 5 different products, and 15 genes are part of the BGC comprising *sven0503* to *sven0517*. The five products are formed from chorismic acid by several reactions involving several of the so-called Pch proteins.

**FIGURE 3:**
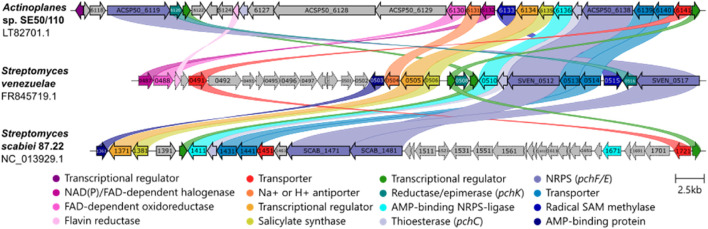
Watasemycin biosynthetic gene cluster comparison of different species. **(A)** *Actinoplanes* sp. SE50/110 (LT827010.1), **(B)** *Streptomyces venezuelae* ATCC 10712 (GenBank FR845719.1), and **(C)** *Streptomyces scabiei* 87.22 (NC_013929.1) are compared. Similar colors indicate protein sequence homology. Essential biosynthesis genes are depicted in solid lines for *S. venezuelae* and *S. scabiei*. Clinker was used for analysis with an identity threshold of 0.03% (Gilchrist and Chooi, 2020).

In addition to *S. venezuelae*, aerugine and pyochelin are also naturally produced by *Streptomyces scabies 87.22*. However, watasemycin, thiazostatin, pulicatin, and isopyochelin are not produced by the strain (Liu J. et al., 2021). A cluster comparison of these three strains revealed a conserved genomic region of biosynthesis genes (Figure 3). *S. scabies* carries 13 genes (*scab1361–1481*) that are associated with aerugine and pyochelin production. Except for *scab_1391*, *Actinoplanes* sp. SE50/110 encodes all homologous genes of all essential *S. scabies* biosynthesis genes within BGC 15. Since *S. scabies* produces two of five products from a similar pathway in *S. venezuelae*, the smaller BGC is no surprise. *S. scabiei* misses *sven0504* and *sven0515* homologs, coding for Na^+^ H^+^ antiporter (PF00999) and an SAM methylase protein, which is essential for methylation of thiazostatin to watasemycin in *S. venezuelae*. Interestingly, *S. scabiei* does not carry any for the thiazoline-reducing *pchK* homologs. However, putative oxidoreductase coding *scab_1461* is homologous to thiazolinyl reductase *pchG*, which performs the reducing step in the pyochelin biosynthesis of the first known pyochelin producer *Pseudomonas aeruginosa* (Reimmann et al., 2001). Thus, it is possible that *S. scabiei pchG* (*scab_1461)* takes over the essential function of *pchK*. *S. venezuelae* carries two *pchK* genes, *sven0508* and *sven0516*, whereas only *sven0516* is essential for thiazoline reduction catalysis within the biosynthesis (Inahashi et al., 2017). *Actinoplanes* sp. SE50/110 only carries the homolog (*ACSP50_6120)* of the functional *sven0516.* When the gene cluster is compared to *S. venezuelae*, it encodes homologs of all biosynthesis-associated genes, except a *sven0503*, which codes for an AMP-binding domain (Pfam:PF00501) containing protein of an unknown function.

The gene cluster is conserved among these three strains. However, *Actinoplanes* sp. SE50/110 homologs of essential biosynthesis genes *pchF* (*ACSP50_6119*), *pchK* (*ACSP50_6120*), and transcriptional regulator (*ACSP50_6121*) are coded in greater distance to all other suspected biosynthesis genes, with several genes in between. Regarding a high structural similarity of compounds 6 and 7 to the five known pathway products, these cluster arrangements could indicate an extended biosynthesis pathway. The genes, which are coded in between the putative gene cluster, code for polyketide synthases (PKSs) (*ACSP50_6127, ACSP50_6128, ACSP50_6129)*, flavin reductase (NADP(H)) (*ACSP50_6125)*, tetracycline 7-halogenase (*ACSP50_6130),* and Na+/H+ antiporter (*ACSP50_6131, sven0504)* proteins. These genes belong to a subcluster comprising *ACSP50_6125*–*ACSP50_6132,* with 50% similarity the neocarzilin A/B biosynthetic gene cluster (BGC0000111) of *Streptomyces carzinostaticus.*

*Actinoplanes* sp. SE50/110 carries with BGC 15 (Table 1) a cluster that has high similarity to the BGC responsible for the biosynthesis of the mentioned molecules in *S. scabies* and *S. venezuelae*. Nevertheless, the cluster has differences to the other BGCs, with the *S. venezuelae pchK* homolog and *S. scabies pchG* homolog missing. It was further shown that the BGC 15 contains further putative biosynthetic genes that are in between the otherwise compact homologous cluster. These genetic variations of the BGC suggest possible extensions or modifications in the biosynthetic pathway in *Actinoplanes* sp. SE50/110, where additional genes for polyketide synthases and other enzymes may contribute to producing structurally related compounds. Despite the high similarity of the BGC 15 to the watasemycin BGC of *S. venezuelae* and pyochelin BGC of *S. scabies*, it must be further investigated to confirm whether BGC 15 is responsible for the production of the identified molecules in *Actinoplanes* sp. SE50/110.

### 3.4 Identification of thiazostatin D and thiazostatin E as novel thiazostatin derivates with anti-cancer activity

Beside the five identified products of the watasemycin biosynthesis, two similar compounds were detected: compound 6 with *m/z* 369.0929 [M + H]^+^ and compound 7 with *m/z* 355.0779 [M + H]^+^. Calculated elemental composition is C_16_H_20_N_2_O_4_S_2_ for compound 6 and C_15_H_18_N_2_O_4_S_2_ for compound 7. Since the molecular mass and elemental composition are similar to those of the prior mentioned molecules, an association to the watasemycin biosynthesis was suspected.

The compound was isolated as described above using the [M + H]^+^ signal *m/z* 35,518 (low-resolution MS). Within the second isolation round, compound 7 eluted in an overlapping peak. Longer gradients did not lead to a better separation of the two species of the compound. The fractions were pooled and reduced to dryness with the weight of 1.0 mg.

**FIGURE 4:**
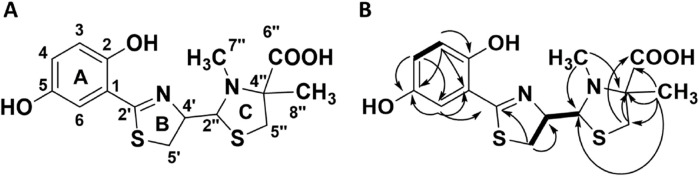
**(A)** The elucidated molecular scaffold of thiazostatin D (7a) and thiazostatin E (7b). **(B)** The HMBC (arrows) and COSY (bold lines) used to elucidate the structure of the molecular scaffold.

Although compound 7 was repeatedly isolated as a single peak by mass-guided preparative HPLC, the results of analyzing the NMR spectra of 7 showed that it consisted of two structurally closely related compounds. This was apparent as there were double signals for most of the carbon and hydrogen atoms. There was no indication of rotamers. Due to the similarity to the thiazostatins, which did not produce rotamers but thus existed as two stereoisomers in position 2″ (thiazostatin A and B), it was hypothesized that the two compounds are stereoisomers. Structure elucidation was conducted using the mixed sample, as the two constituents, now termed 7a and 7b, could not be separated. 1D (1H and 13C; Table 3; Supplementary Figures S9, S10) and 2D (HSQC, HMBC, COSY, ROESY, and H2BC; Figure 3B; Supplementary Figures S11–S15) data of the 7a/7b mixture confirmed their structural similarity to the previously reported compounds in the thiazostatin and watasemycin families (Supplementary Figure S8). The connectivities of the atoms of the B and C rings were determined through HMBC and COSY signals (Figures 1B, Supplementary Figure S4, S8) and by comparing NMR data with those recorded for the previously recorded thiazostatin and watasemycin variants (Shindo et al., 1989). This leaves a C_6_H_5_O_2_ fragment to be assigned, indicative of the presence of a benzene ring substituted with two hydroxy groups. The signals of the benzene ring were indeed detected. A clear HMBC was visible from an aromatic carbon signal (H-6, δH 6.82–6.75 in both 7a and 7b) to the C-2’ (δC 170.5 in 7a, δC 171.0 in 7b) carbon atom, linking the benzene fragment to the rest of the molecule. In addition to CH-6 (δC 114.7 and 114.9), two distinct signals for aromatic CH-groups (CH-3, δC 117.4 in both 7a and 7b, CH-4, δC 121.0 in 7a, and δC 121.1 in 7b), a fully substituted aromatic carbon atom (C^−1^, δC 115.3 in both 7a and 7b), and two distinct signals for hydroxylated aromatic carbon atoms (C-2, δC 151.3 and C-5, δC 149.5 in both 7a and 7b, Supplementary Figure S12) were visible, confirming the presence of a dihydroxybenzene fragment. The coupling patterns of the benzylic protons (Supplementary Figure S9) revealed the substitution pattern to be 2-OH and 5-OH based on that the assigned proton atom H-6 was a double with a small coupling, characteristic for an isolated proton with a weak meta-coupling, in this case to proton atom H-4. Proton atom H-4 was a doublet of a doublet with one strong 8 Hz ortho-coupling to proton atom H-3 and one weak meta-coupling to proton atom H-6. The lack of a methylation in the C-5 position makes the compounds more similar to the thiazostatins rather than the C-5 methylated watasemycins. The compounds were consequently given the names thiazostatin D (7a) and thiazostatin E (7b) since Liu et al. (2022) published a thiazostatin-like compound bearing one hydroxyl group at ring A in position 2 and O-methylation of the carboxylic acid on ring C. The difference between 7a and 7b is attributed to different configurations in the C-2″ position, as for thiazostatins A and B (Figure 4).

**TABLE 3:** 1H and 13C NMR assignments for the two constituents isolated together in sample 7 (1H 600 MHz, 13C 150 MHz, DMSO-d_6_).

	Thiazostatin C (7a)		Thiazostatin D (7b)	
position	δ_C_, type	δ_H_ (*J* in Hz)	δ_C_, type	δ_H_ (*J* in Hz)
1	115.3, C		115.3, C	
2	151.3, C		151.3, C	
3	117.4, CH	6.82–6.75, m^a^	117.4, CH	6.82–6.75, m^a^
4	121.0, CH	6.88–6.82, m^a^	121.1, CH	6.88–6.82, m^a^
5	149.5, C		149.5, C	
6	114.7**, CH	6.82–6.75, m^a^	114.9**, CH	6.82–6.75, m^a^
2′	170.5, C		171.0, C	
4′	79.0, CH	5.28, td (9.1, 4.0)	78.2, CH	5.14, td (9.0, 3.7)
5′a	32.8, CH_2_	3.57, dd (11.0, 9.2)	31.1, CH_2_	3.82, m
5′b		3.19, m^a^		3.37, m
2″	73.4, CH	4.57, d (3.8)	72.5, CH	4.52, d (3.8)
4″	73.1, C		72.7, CH	
5″	39.0, CH_2_	3.14, m	38.9, CH_2_	3.19, m^a^
6″	174.9, C		174.2, C	
7″	33.7, CH_3_	2.51, s	34.5, CH_3_	2.39, s
8″	22.7, CH_3_	1.37, s	14.1, CH_3_	1.26, s

^a^ signals are overlapping, **unclear if the measured signal belongs to 7a or 7b.

### 3.5 Bioactivity of the isolated compound

**FIGURE 5:**
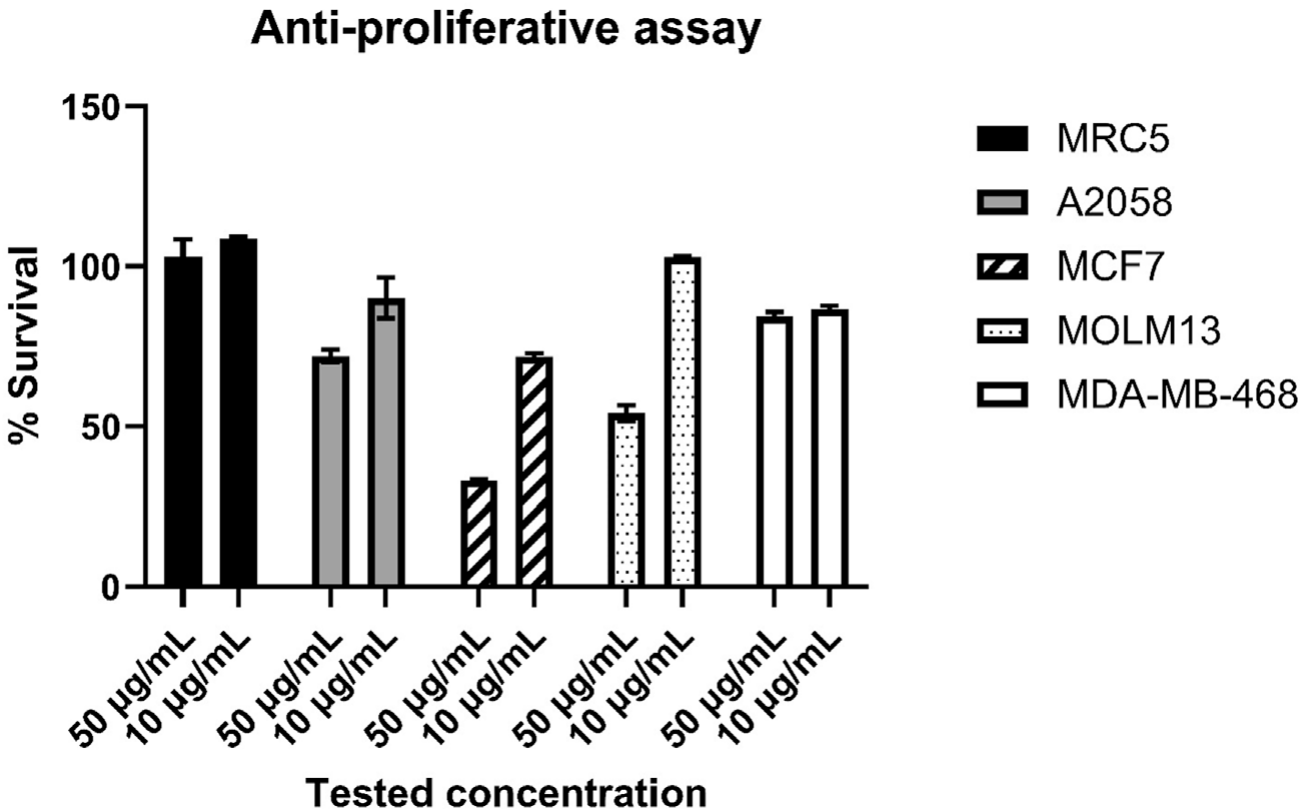
Results for the anti-proliferative assay, the thiazostatin mixture was tested at 50 and 10 μg·mL^−1^ against the five cell-lines MRC5 (lung fibroblast), A2058 (melanoma), MCF7 (breast cancer), Molm13 (acute myeloid leukemia), and MDA-MB-468 (mammary gland, epithelial adenocarcinoma). The results are given as relative/normalized results (% survival) in relation to the untreated control (100% growth).

The isolated compounds were a mixture of the two closely related thiazostatins D and E, and since a further separation of the two isomers was not possible, the two compounds were tested for potential bioactivities. For the MIC assay, we selected *S. aureus* and *E. coli* as Gram-positive and Gram-negative pathogens, respectively, as well as the Gram-positive *S. agalactiae* since the latter one has shown the highest sensitivity when screening extracts, extract fractions, and compounds for anti-microbial activities in our experience (Kristoffersen et al., 2018; Schneider et al., 2019; Schneider et al., 2020). We have tested the compound mixture for potential bioactivity against the three bacteria at 100 μg·mL^−1^ test concentration and did not observe an anti-microbial effect. For the anti-cancer assay, we have initially tested the compound mixture at 100 μg·mL^−1^ against the three cell lines MCF7, MRC5, and A2058, where A2058 has shown 53% survival, MCF7 has shown 15% survival, and MRC5 has shown 101% survival; since the immortalized lung-fibroblast cell line MRC5 was seemingly not affected by the compounds and the MCF7 (breast cancer) cell line has shown just 15% survival, we repeated the anti-proliferative assay using five cell lines (the three initially tested cell lines plus Molm13 and MDA-MB-468) and concentrations of 50 μg·mL^−1^ and 10 μg·mL^−1^, the limitation to five cell lines and two concentrations was due to the available compound quantity. The results are given in Figure 5.

### 3.6 Different σ-factor engineering approaches facilitate the production shift within the pathway and enable activation of the production of a novel compound

To investigate the influence of *Actinoplanes* sp. SE50/110 σ factor and σ factor-related genes on metabolite production, alternative σ factor encoding *sigH* and anti-anti-σ factor encoding *ACSP50_0284* (0284) were manipulated as examples. Schlüter *et al.* showed that *sigH* overexpression enhanced acarbose production *Actinoplanes* sp. SE50/110, making it an interesting gene target to investigate its influence on the production of further metabolites (Schlüter et al., 2024). *ACSP50_0284* encodes a sulfate transporter and anti-sigma-factor antagonist (STAS) domain protein (Kormanec et al., 2016; Mingyar et al., 2014; Sevcikova et al., 2010; Sevcikova et al., 2021). Gene deletion and expression strains were created for both genes, and the production of the different compounds was investigated since preliminary data indicated a putative regulatory influence of these genes on the production of further metabolites.

**FIGURE 6:**
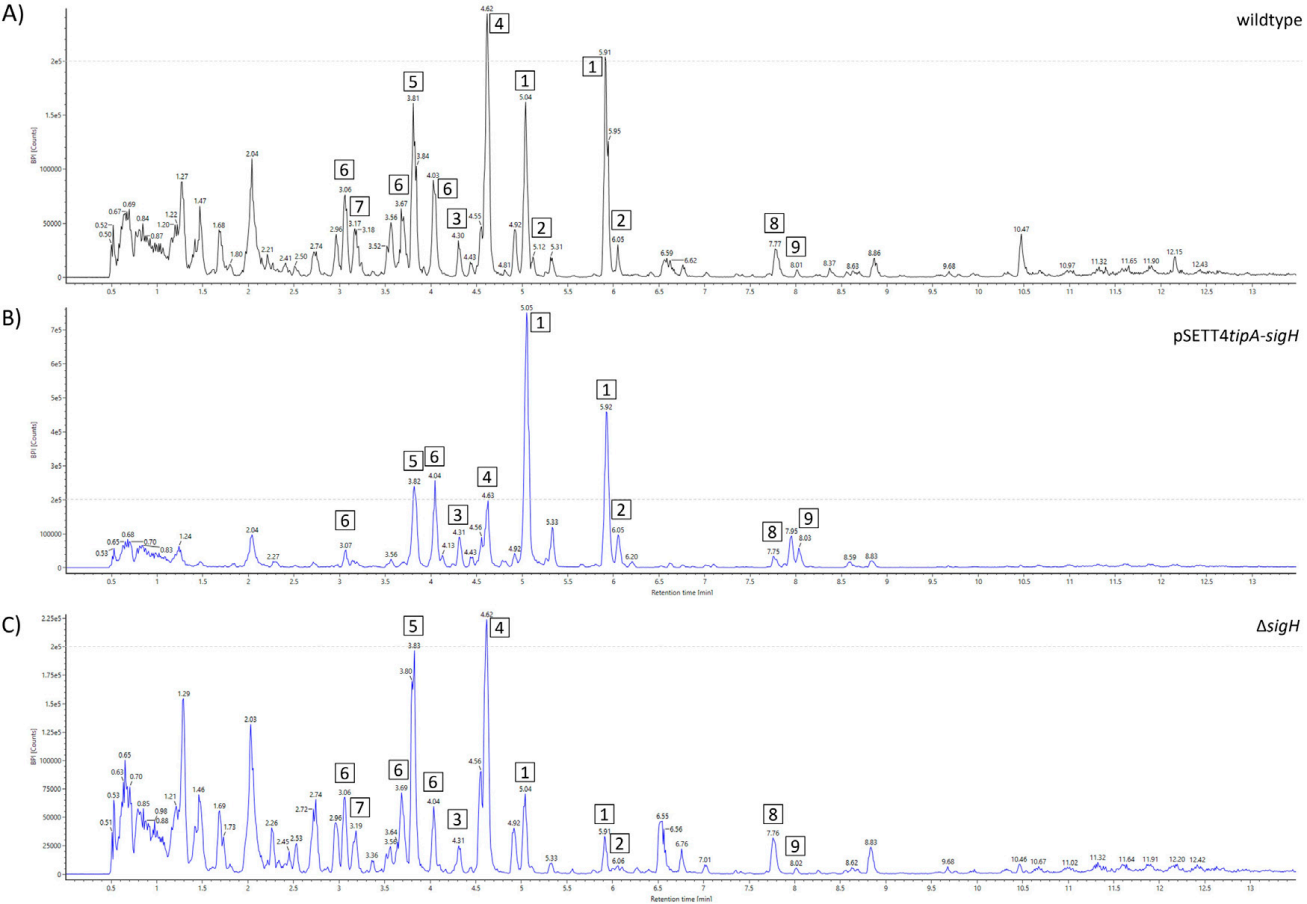
Metabolite spectrum of *Actinoplanes* sp. SE50/110 *sigH* mutant strains. BPI chromatograms of the whole-cell extract using UHPLC-QToF-MS. Peak intensities are shown for the **(A)** wildtype, **(B)** *sigH* expression strain (pSETT4*tipA-sigH*), and **(C)** *sigH* deletion strain (*ΔsigH*). Different compounds are highlighted: 1) watasemycin *m/z* 353.0988 [M + H]^+^, 2) thiazostatin *m/z* 339.0836 [M + H]^+^, 3) isopyochelin *m/z* 325.0656 [M + H] ^+^, 4) pulicatin *m/z* 324.0743 [M + H]^+^, 5) aerugine *m/z* 210.0583 [M + H]^+^, 6) *m/z* 369.0929 [M + H]^+^, 7) thiazostatin D/E *m/z* 355.0779 [M + H] ^+^, 8) *m/z* 355.0779 [M + H]^+^, and 9) *m/z* 440.2768 [M + H]^+^.

*SigH* expression resulted in an increased peak intensity of watasemycin (1) and putative watasemycin-related compound 6 of *m/z* 369.0929 [M + H]^+^ when compared to the wildtype (Figure 6). While the peak intensity of pulicatin (4) was decreased, the peak intensity of the highly similar aerugine (5) was slightly increased. The previous sections identified that watasemycin pathway-related thiazostatin D (7a) and thiazostatin E (7b) could not be detected in the sample, indicating the prevention of its production due to the gene expression. Surprisingly, the *sigH* deletion strain (Δ*sigH*) did not show the complete opposite effect of gene overexpression but led to a decrease in the watasemycin peak intensity, without further influence on pulicatin, aerugine, and thiazostatin D/E peak intensity. The Δ*sigH* strain showed an opposite effect on the peak intensities of the 2-hydroxyphenylthiazoline family compounds. Under gene deletion, watasemycin (1) peak intensity is significantly reduced in relation to the other watasemycin pathway-derived compounds and the peak intensity of aerugine (5) and pulicatin (4) is increased. This indicates a putative regulatory influence of σH on this biosynthesis pathway. Since the peak intensity of thiazostatin D/E (7) is also affected, its origin from the same biosynthesis pathway is even more assumed.

**FIGURE 7:**
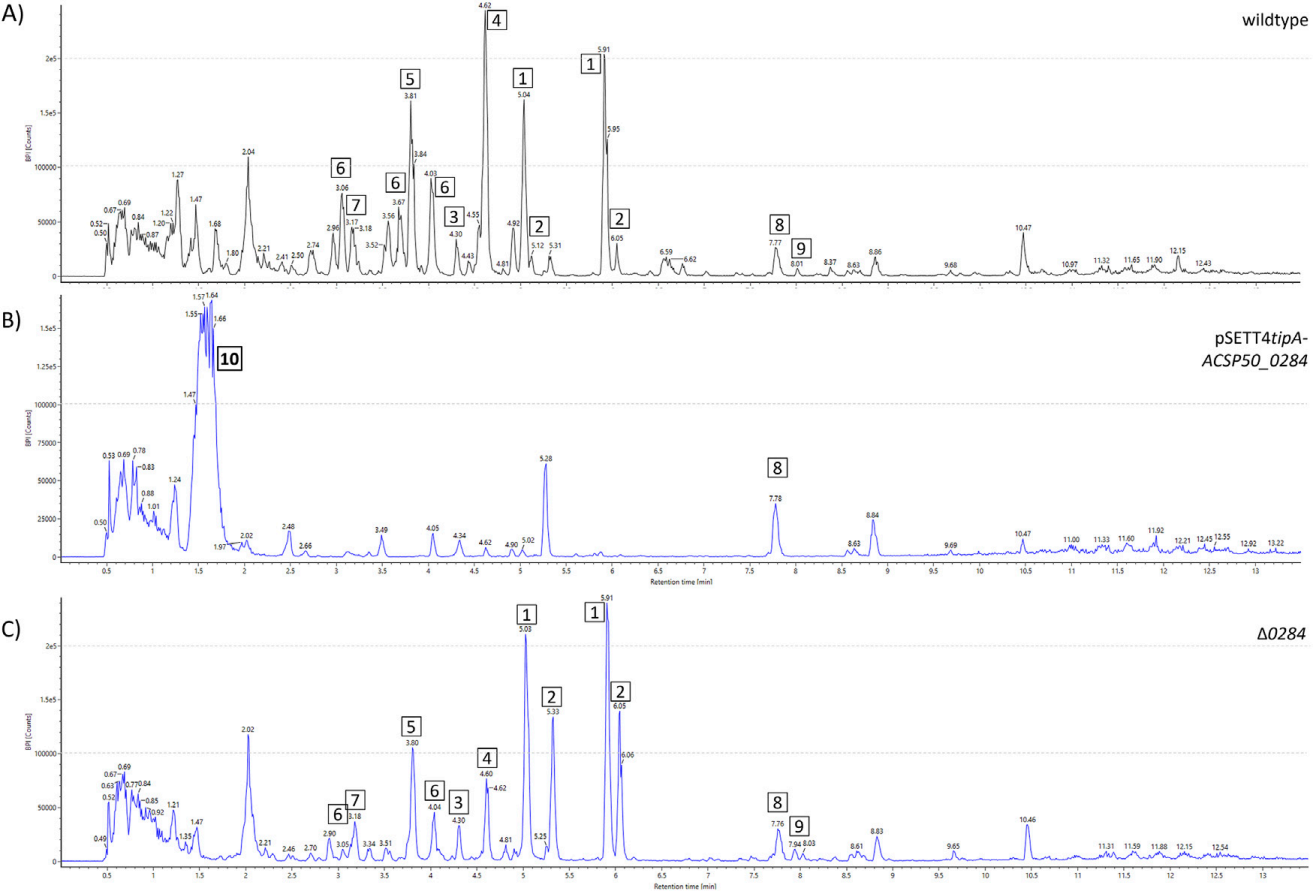
Metabolite spectrum of *Actinoplanes* sp. SE50/110 *ACSP50_0284* mutant strains. BPI chromatograms of the whole-cell extract using UHPLCQToF-MS Peak intensities are shown for the **(A)** wildtype, **(B)** *ACSP50_0284* expression strain (pSETT4*tipA-ACSP50_0284*), and **(C)** *ACSP50_0284* deletion strain(Δ0284). Different compounds are highlighted: 1) watasemycin *m/z* 353.0988 [M + H]^+^, 2) thiazostatin *m/z* 339.0836 [M + H]^+^, 3) isopyochelin *m/z* 325.0656 [M + H]^+^, 4) pulicatin *m/z* 324.0743 [M + H]^+^, 5) aerugine *m/z* 210.0583 [M + H]^+^, 6) *m/z* 369.0929 [M + H]^+^, 7) thiazostatin D/E *m/z* 355.0779 [M + H]^+^, 8) *m/z* 355.0779 [M + H]^+^, 9) *m/z* 440.2768 [M + H]^+^, and 10) *m/z* 462.1643 [M + H]^+^.

Interestingly, *ACSP50_0284* manipulation had a more severe effect on the watasemycin biosynthesis pathway and its putative products (Figure 7). While *ACSP50_0284* deletion (Δ*0284*) resulted in a change in the peak intensity distribution of the 2-hydroxyphenylthiazoline family molecules with an increased peak intensity of thiazostatin (2) and reduced peak intensity of pulicatin (4), *ACSP50_0284* expression prevented the production of all products. These were not detectable in the whole-cell extract of the *ACSP50_0284* expression strain. Strikingly, we hereby observed the production of an unknown compound 10 of *m/z* 462.1643 [M + H]^+^ with calculated elemental composition C_19_H_27_NO_12_. This highly polar compound was not detected in the *Actinoplanes* sp. SE50/110 wildtype and is exclusively produced by the *ACSP50_0284* expression strain. Despite several purification attempts, sufficient purity of the isolate was not achieved and prevented structure elucidation.

**FIGURE 8:**
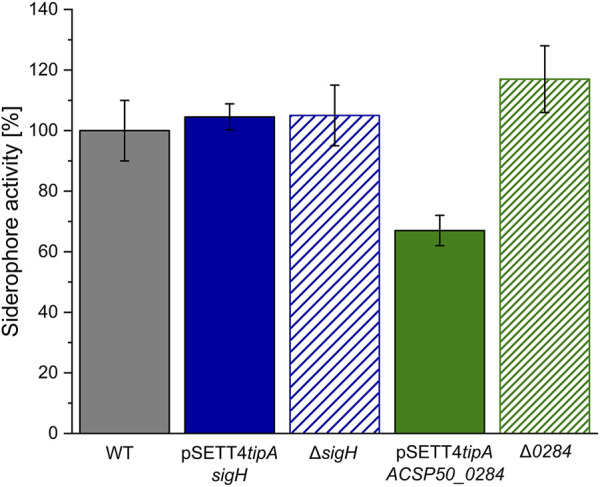
Siderophore activity of *Actinoplanes* sp. SE50/110 mutant strains (n = 3). The chrome Azurol S (CAS) assay was performed for the wildtype (WT), *sigH* or *ACSP50_0284* expression strains pSETT4*tipA-sigH* and pSETT4*tipA-ACSP50_0284,* and gene deletion strains *∆sigH* and *∆0284*.

The rendered product distribution of the identified compounds by sigma factor engineering is suspected to affect the strains’ siderophoric ability since aerugine and (iso)pyochelin have siderophore and iron-chelating ability (Inahashi et al., 2017) and thiazostatin and watasemycin are suspected of having siderophore activity according to the functional group-based prediction provided by SIDERITE (He et al., 2024). A colorimetric-based Chrome Azurol S (CAS) assay was performed with supernatant. The *sigH* expression and deletion strains had a similar siderophore activity than the wildtype (Figure 8). Its gene manipulation resulted in a shift of peak intensities between the pathway products, but no drastic overall change in siderophore activity, whereas the expression of *ACPS50_0284* resulted in the complete production shutdown of the watasemycin pathway products and the significant reduction to 68% of siderophore activity within the *Actinoplanes* sp. SE50/110 supernatant. Interestingly, the deletion strain ∆0284 showed a slightly increased, but insignificant, siderophore activity of 117% wildtype activity.

## 4 Discussion

In this study, we describe the identification of five thiazostatin-related molecules and the discovery, identification, and characterization of similar compounds that were termed thiazostatin D and thiazostatin E. We further describe the influence of sigma factor engineering on the production of these compounds that led to changes in peak intensities between them and the exclusive production of a novel unidentified compound.

Thiazostatin D and thiazostatin E have a high similarity to thiazostatin A and B that did not show antimicrobial activity against Gram-positive and Gram-negative bacteria as well as fungi (Shindo et al., 1989). No bioactivity of thiazostatin C was shown so far (Liu et al., 2022). To our knowledge, thiazostatins were never tested against cancer cells. While lung fibroblasts were not affected by tested concentrations of 50 μg and 10 µg thiazostatin D/E, the breast cancer cell line (MCF7) has shown the highest sensitivity (Figure 5). The tested concentrations of 50 μg·mL^−1^–100 μg·mL^−1^ that showed activity are relatively high (Kristoffersen et al., 2018; Schneider et al., 2019). Furthermore, since a mixture of two close structural isomers was tested, it is unknown whether both compounds have the same level on bioactivity as it was shown that watasemycin A has a stronger antibacterial activity than watasemycin B. At 0.39 μg·mL^−1^, watasemycin A shows activity against Gram-negative strain *Proteus mirabilis* ATCC 21100 and at 12.5 μg·mL^−1^ and 25.0 μg·mL^−1^ against Gram-positive bacteria at against *Staphylococcus aureus* 209P JC^−1^ and *Bacillus subtilis* ATCC 6633, while its isomer watasemycin B has antibacterial activity against these strains at 6.25 μg·mL^−1^, 25.0 μg·mL^−1^, and 50 μg·mL^−1^ activity (Sasaki et al., 2002).

Under *sigH* expression, no significant amounts of the thiazostatins were produced. However, compound 6 was still produced, despite the assumption that both compounds are produced by the same pathway since the predicted elemental compositions of C_16_H_20_N_2_O_4_S_2_ (compound 6) and C_15_H_18_N_2_O_4_S_2_ (compound 7) are highly similar. They differ in CH_2_ with a weight difference of 14 kDa, indicating the methylation of thiazostatin D/E as the final molecular modification for the formation of compound 6. However, the results of the *sigH* expression indicate that production of compound 6 may not rely on thiazostatin D/E as the precursor and could be based on watasemycin. Gene deletion of either *sigH* or *ACSP50_0284* further shows that both influence the production of the pathway products by redirecting the metabolic flux toward individual products. It further shows that both proteins are not essential for the pathway activity. However, as shown for *ACSP50_0284*, its overexpression can be disadvantageous for the cell and prevent product formation. Its regulatory mechanism on this biosynthesis pathway and why overexpression results in the pathway inhibition must be elucidated in further studies.

The *sigH* expression and deletion strains show a similar siderophore activity to the wildtype. This was expected since some products of the watasemycin pathway have siderophore activity (He et al., 2024) and σ factor manipulation resulted in a shift of peak intensities. A reduced siderophore activity under *ACSP50_0284* expression proves that the products of the watasemycin biosynthesis significantly contribute to the siderophore activity of *Actinoplanes* sp. SE50/110. That the siderophore activity was not eliminated proves that the strain produces further iron-chelating molecules, which are detected since the assay detects any siderophore activity in the supernatant. This aligns with two further gene clusters for the production of putative siderophores that were predicted for *Actinoplanes* sp. SE50/110. Thereby, cluster 18 shows a high identity of 75% to the siderophore desferrioxamine E (Table 1).

While *ACSP50_0284* prevented biosynthesis of compounds 1–7, a large peak of a new compound pf *m/z* 462.1643 [M + H]^+^ was detected. The compound elutes early at 1.47–1.68 min and must be highly polar. Its elemental composition was predicted as C_19_H_27_NO_12,_ and no similar molecules could be determined during comprehensive literature and database research, indicating that compound 10 could be a novel molecule. The *ACSP50_0284* expression strain exclusively produces the compound, which is not detectable in any other *Actinoplanes* sp. SE50/110 wildtype or mutant strain. We conclude that its gene expression facilitates the activation of a silent gene cluster. Silent gene clusters are a major challenge in natural product discovery, with the majority of *Streptomyces* BGCs being silent or cryptic, complicating natural product discovery (Liu Z. et al., 2021). For the activation of silent BGCs, targeted (Bunet et al., 2011; Laureti et al., 2011; Lin et al., 2006; Myronovskyi and Luzhetskyy, 2016; Rutledge and Challis, 2015; Sidda et al., 2014), untargeted (Abdel-Razek et al., 2018; Daniel-Ivad et al., 2017; Hemphill et al., 2017; Tanaka et al., 2010), or semi-targeted approaches (Mingyar et al., 2021) can be applied. It was previously shown that the manipulation of master regulators (Daniel-Ivad et al., 2017; Yushchuk et al., 2021) can activate silent gene clusters. Despite encoding an anti-anti-σ factor, which is no direct transcriptional activator, it can confer the σ factor. It is fascinating that the ASCP50_0284 protein does not directly activate BGC expression but interferes in a complex regulatory network that affects the product formation. We hereby showed that not only the manipulation of a transcriptional regulator but also the regulatory protein for the antagonist of a transcription factor can facilitate the activation of a silent gene cluster. To the best of knowledge, this was not shown before for an anti-anti-σ factor.

## Data Availability

The complete genome sequence of Actinoplanes sp. SE50/11 is available through Gene Bank, ID: LT827010.1, the sequences of the oligonucleotides used within this study can be found within the Supplementary Information.
