# Supplementary material for: Discovery of thiazostatin D/E using UPLC-HR-MS2-based metabolomics and σ-factor engineering of Actinoplanes sp. SE50/110
Source: Front Bioeng Biotechnol. 2024 Nov 25;12:1497138. doi: 10.3389/fbioe.2024.1497138 (PMC11626248; doi:10.3389/fbioe.2024.1497138)
Supplement: Supplementary file 1 [file DataSheet1.PDF]

Discovery of thiazostatin D/E produced by *Actinoplanes* sp.SE50/110 and σ factor engineering mediated production manipulation of five 2-hydroxyphenylthiazoline family molecules.

Laura Schlüter[1], Kine Østnes Hansen[2], Johan Isaksson[2], Jeanette Hammer Andersen[3], Espen Holst Hansen[3], Jörn Kalinowski[1,4], Yannik Karl-Heinz Schneider[3*]

[1]Microbial Genomics and Biotechnology, Center for Biotechnology, Bielefeld University, 33594 Bielefeld, Germany

[2]Department of Pharmacy, Faculty of Medicine and Health Sciences, UiT-The Arctic University of Norway, Breivika, N-9037 Tromsø, Norway

[3]Marbio, Faculty for Fisheries, Biosciences and Economy, UiT-The Arctic University of Norway, Breivika, N-9037 Tromsø, Norway

[4]Technology Platform Genomics, Center for Biotechnology, Bielefeld University, 33594 Bielefeld, Germany

Corresponding Author

* Yannik K.-H. Schneider

Supplemental Information Table of Contents

Molecular genetic work

Table S1: Oligonucleotides used in this study

Figure S1: *sigH* (*ACSP50_0507*) expression, based on the integrative pSET4t*ipA*-*sigH* vector, is verified by Nanopore whole genome sequencing.

Figure S2: *sigH* (*ACSP50_0507*) deletion is verified by Nanopore whole genome sequencing.

Figure S3: *ACSP50_0284* expression, based on the integrative pSET4t*ipA*-*sigH* vector, is verified by Nanopore whole genome sequencing.

Figure S4: *ACSP50_0284* deletion is verified by Nanopore whole genome sequencing.

Dereplication

Figure S5. Mass spectra of watasemycin, thiazostatin and isopyochelin.

Figure S6. Mass spectra of pulicatin, aerugine and thiazostatin D/E.

Figure S7. Mass spectra of *m/z* 462.1643 $[M+H]^+$ (compound 10).

NMR Spectroscopic Data for thiazostatin D and E

Figure S8 Structures of the previously reported compounds watasemycin A and B and thiazostatin A and B

Figure S9 ^{1}H NMR (600 MHz, DMSO-d_6) spectrum of thiazostatin D (**7a**) and E (**7b**)

Figure S10 ^{13}C (151 MHz, DMSO-d_6) spectrum of thiazostatin D (**7a**) and E (**7b**)

Figure S11 HSQC + HMBC (600 MHz, DMSO-d_6) spectrum of thiazostatin D (**7a**) and E (**7b**)

Figure S12 Zoom in HSQC + HMBC (600 MHz, DMSO-d_6) spectrum of thiazostatin D (**7a**) and E (**7b**)

Figure S13 COSY (600 MHz, DMSO-d_6) spectrum of thiazostatin D (**7a**) and E (**7b**)

Figure S14 ROESY (600 MHz, DMSO-d_6) spectrum of thiazostatin D (**7a**) and E (**7b**)

Figure S15 H2BC (600 MHz, DMSO-d_6) spectrum of thiazostatin D (**7a**) and E (**7b**)

Table S1: Oligonucleotides used in this study.

**Cloning strategy**	**Name**	**Nucleotide sequence 5‘-3‘**
sigH expression using pSETT4*tipA*	0507_pSETT4_fw	GGCACTAGTCGAGCAACGGAGGTATTCCGATGaccgtcacacagacttcgacg
	0507_pSETT4_rev	GGCGGAAAATCACGCGGCACGAAtcagagcgtctcggcggc
sigH deletion using pCRISPomyces-2: gRNA	gRNA_0507_fw	acgcAACGGCCGAGGGAGTGGGTC
	gRNA_0507_rev	aaacGACCCACTCCCTCGGCCGTT
sigH deletion using pCRISPomyces-2: homologous flanking regions	0507_flank1_fw	TCGGTTGCCGCCGGGCGTTTTTTATcgaggcgcgacgagacttcg
	0507_flank1_rev	cgacgaccgtcgaccgggccgccgagacgctctgag
	0507_flank2_fw	cctcagagcgtctcggcggcccggtcgacggtcgtcgaag
	0507_flank2_rev	GCGGCCTTTTTACGGTTCCTGGCCTcccactgaccgccgaaacg
ACSP50_0284 expression using pSETT4*tipA*	0284_pSETT4_fw	GGCACTAGTCGAGCAACGGAGGTATTCCGATGtccttgacggtgcagaccg
	0284_pSETT4_rev	GGCGGAAAATCACGCGGCACGAAtcagtcgagcgcgggttc
ACSP50_0284 deletion using pCRISPomyces-2: gRNA	gRNA_0284_fw	acgcCCGGACGAAAACGGACAGCC
	gRNA_0284_rev	aaacGGCTGTCCGTTTTCGTCCGG
ACSP50_0284 deletion using pCRISPomyces-2: homologous flanking regions	0284_Flank1_fw	TCGGTTGCCGCCGGGCGTTTTTTATgcatgccgaccacgaccgtc
	0284_Flank1_rev	cctcagcggtgaccgcctcgcgttgttcggtctgcaccg
	0284_Flank2_fw	ggtgcagaccgaacaacgcgaggcggtcaccgctgagg
	0284_Flank2_rev	GCGGCCTTTTTACGGTTCCTGGCCTgggcacggcttcgtgatcg
Screening of the expression plasmid	Screen-pSETT4_fw	TGACCCCATGCCGAACTCAGAAGTGAAACG
	Screen-pSETT4_rev	GTACTTCGTCGTGAAGGTCATGACACCATTATAACGAACG
Screening of the deletion plasmid	Screen_fw	GGCGTTCCTGCAATTCTTAG
	Screen_rev	TCGCCACCTCTGACTTGAGC

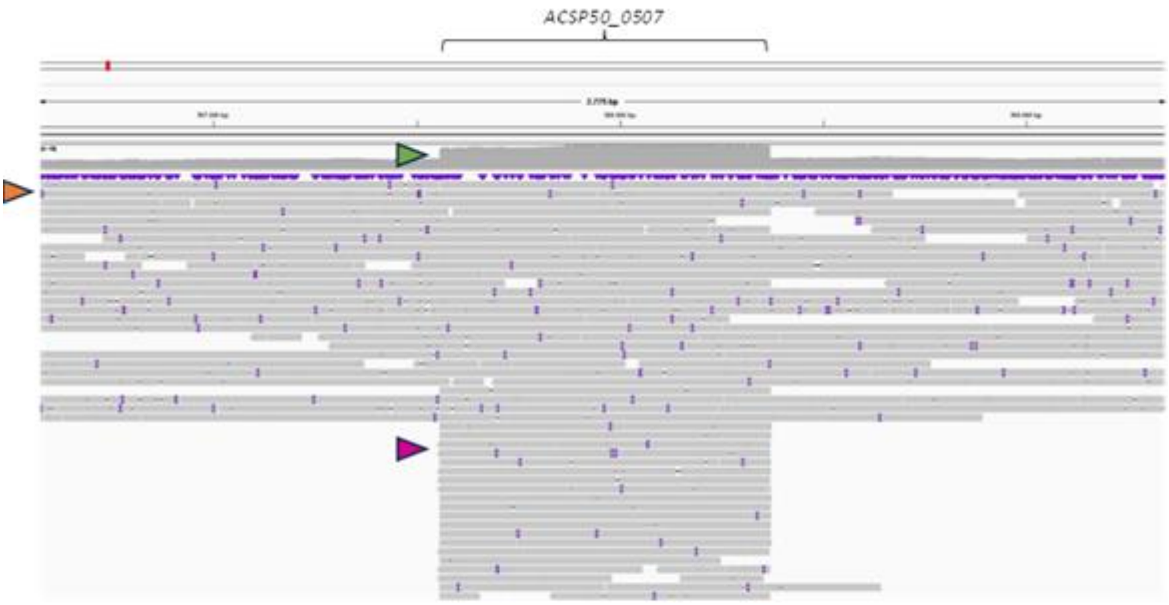

Figure S1: *sigH* (*ACSP50_0507*) expression, based on the integrative pSET4t*ipA*-*sigH* vector, is verified by Nanopore whole genome sequencing. IGV (Robinson et al. 2011) view shows an increased number of reads at the gene region of *sigH*, due to the integration of a second gene copy (green arrow). Presence of the chromosomal gene copy is confirmed by reads overlapping the gene environment (orange arrow) and the additional gene copy is confirmed by reads aligning to *sigH* but without any overlap to the genetic surrounding (pink arrow). Purple lines indicate insertions, which are caused by increased error rates during Nanopore sequencing due to homobase polymers. Reads were mapped using minimap2 (Li 2021), sorting and indexing was performed using samtools (Danecek et al. 2021).

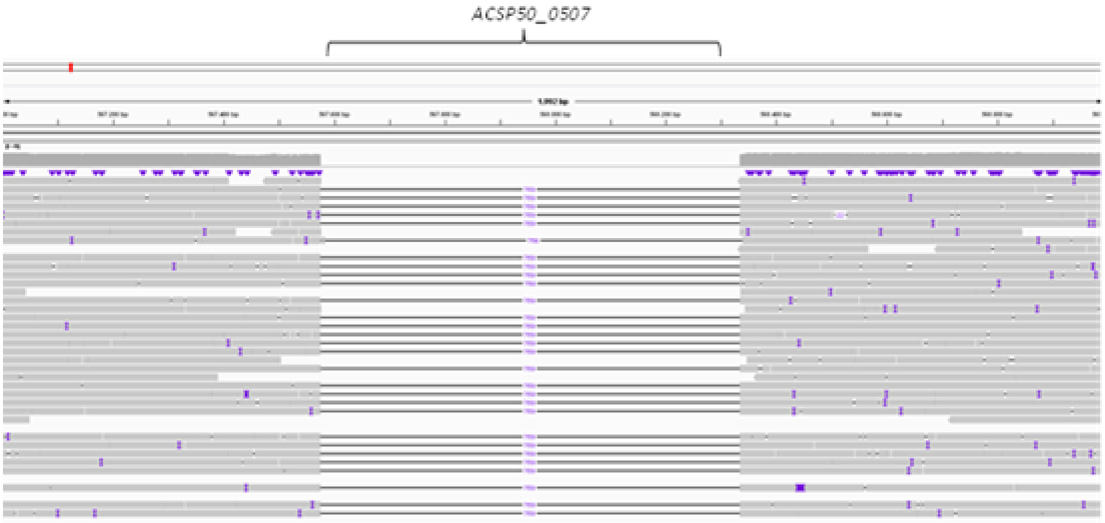

Figure S2: *sigH* (*ACSP50_0507*) deletion is verified by Nanopore whole genome sequencing. Integrative Genomics Viewer (IGV) (Robinson et al. 2011) view of a sequence deletion of base pairs within the gene region of *sigH* is shown. Purple lines indicate insertions, which are caused by increased error rates during Nanopore sequencing due to homobase polymers. Reads were mapped using minimap2 (Li 2021), sorting and indexing was performed using samtools (Danecek et al. 2021).

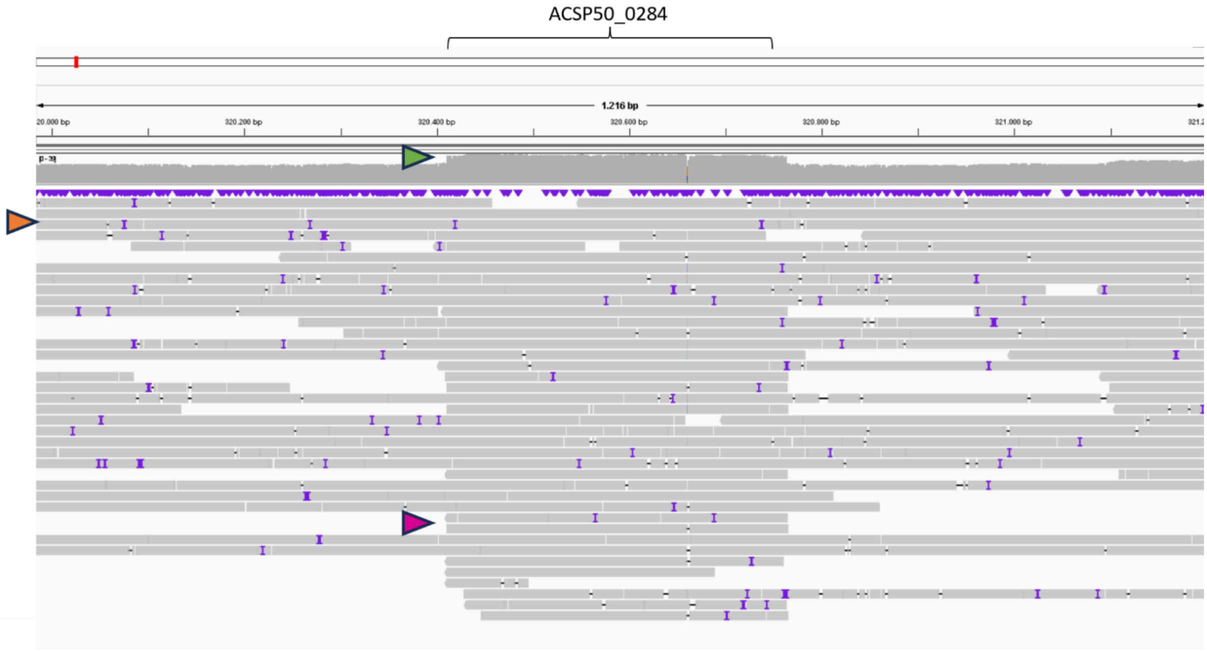

Figure S3: *ACSP50_0284* expression, based on the integrative pSET4t*ipA*-*ACSP50_0284* vector, is verified by Nanopore whole genome sequencing. IGV (Robinson et al. 2011) view shows an increased number of reads at the gene region of *ACSP50_0284*, due to the integration of a second gene copy (green arrow). Presence of the chromosomal gene copy is confirmed by reads overlapping the gene environment (orange arrow) and the additional gene copy is confirmed by reads aligning to *ACSP50_0284* but without any overlap to the genetic surrounding (pink arrow). Purple lines indicate insertions, which are caused by increased error rates during Nanopore sequencing due to homobase polymers. Reads were mapped using minimap2 (Li 2021), sorting and indexing was performed using samtools (Danecek et al. 2021).

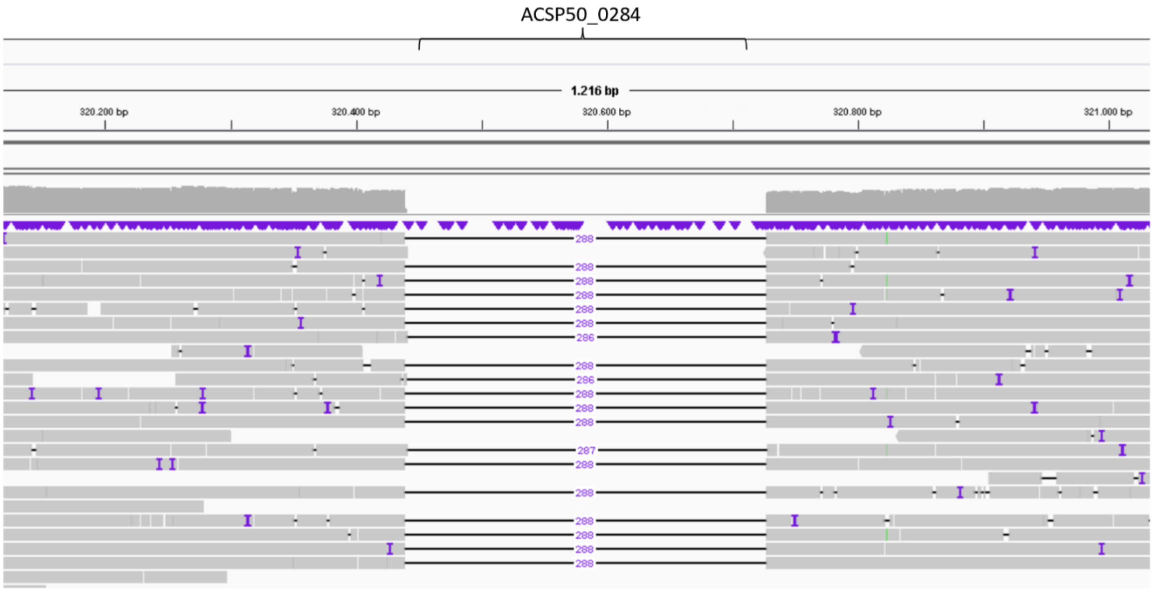

Figure S4: *ACSP50_0284* deletion is verified by Nanopore whole genome sequencing. Integrative Genomics Viewer (IGV) (Robinson et al. 2011) view of a sequence deletion of base pairs within the gene region of *ACSP50_0284* is shown. Purple lines indicate insertions, which are caused by increased error rates during Nanopore sequencing due to homobase polymers. Reads were mapped using minimap2 (Li 2021), sorting and indexing was performed using samtools (Danecek et al. 2021).

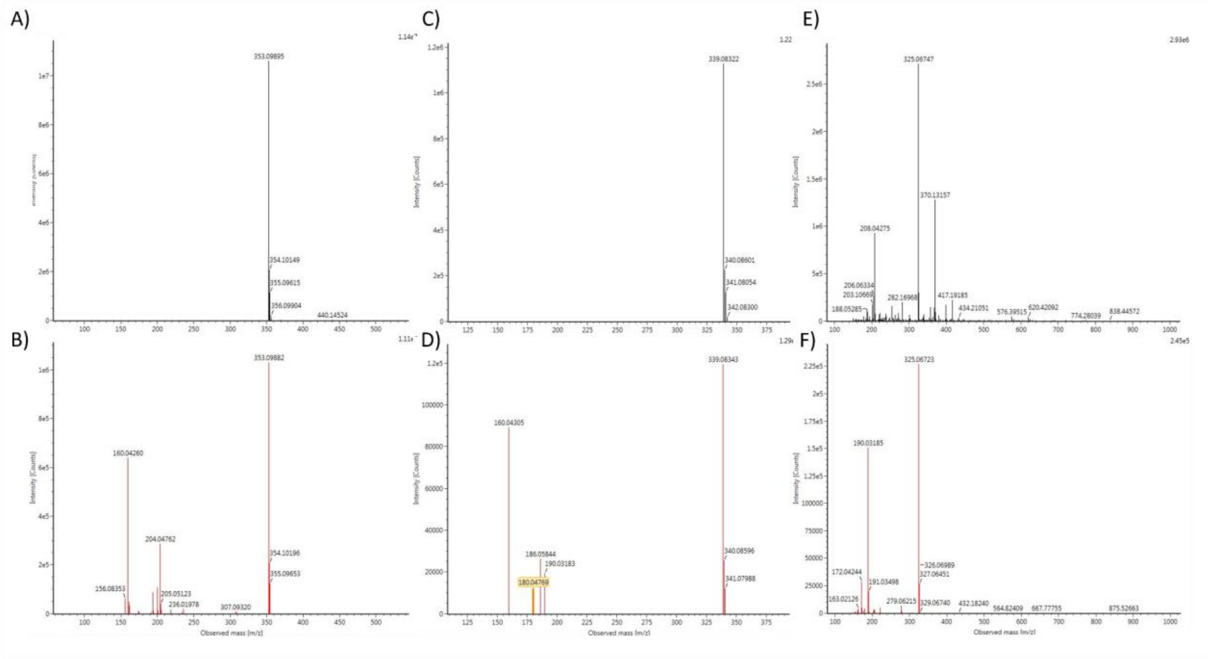

Figure S5. Mass spectra of watasemycin, thiazostatin and isopyochelin. The low-collision energy spectra, determined in the ESI+ mode, is given in black and the high-collision energy spectra (20-80 eV ramp) in red. A,B) watasemycin (compound 1) with *m/z* 353.0988 , C,D) thiazostatin (compound 2) with *m/z* 339.0836, E,F) isopyochelin (compound 3) with *m/z* 325.0656.

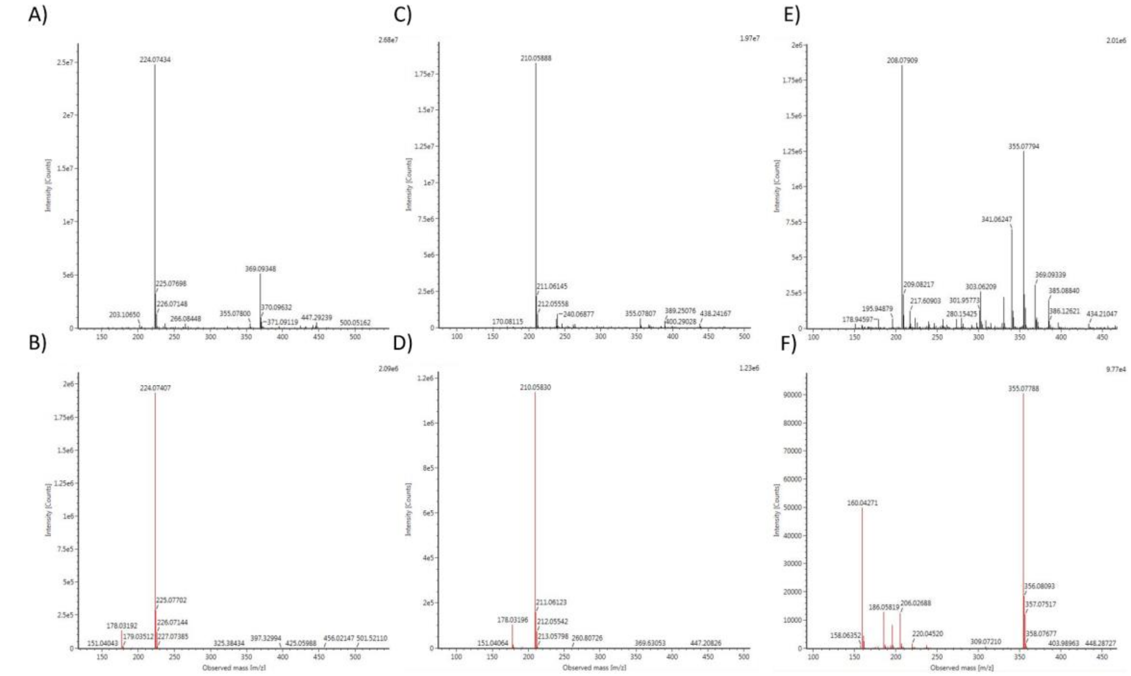

Figure S6. Mass spectra of pulicatin, aerugine and thiazostatin D/E. The low-collision energy spectra, determined in the ESI+ mode, is given in black and the high-collision energy spectra (20-80 eV ramp) in red. A,B) pulicatin (compound 4) with *m/z* 224.0743 , C,D) aerugine (compound 5) with *m/z* 210.0583, E,F) thiazostatin D/E (compound 7a/b) with *m/z* 355.0779.

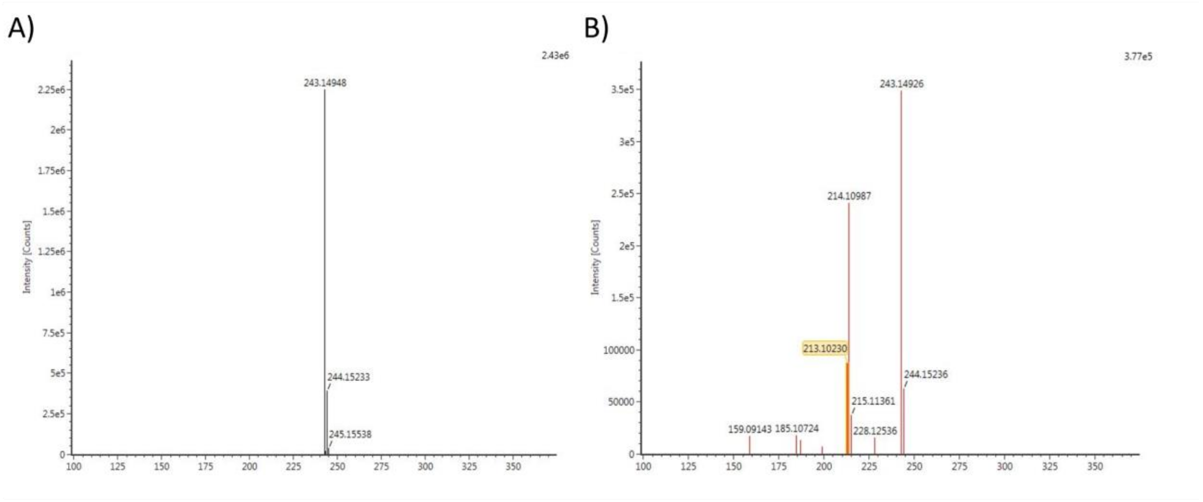

Figure S7. Mass spectra of *m/z* 462.1643 $[M+H]^+$ (compound 10). A) The low-collision energy spectra, determined in the ESI+ mode, is given in black and B) the high-collision energy spectra (20-80 eV ramp) in red. The compound is exclusively produced under *ACSP50_0284* expression.

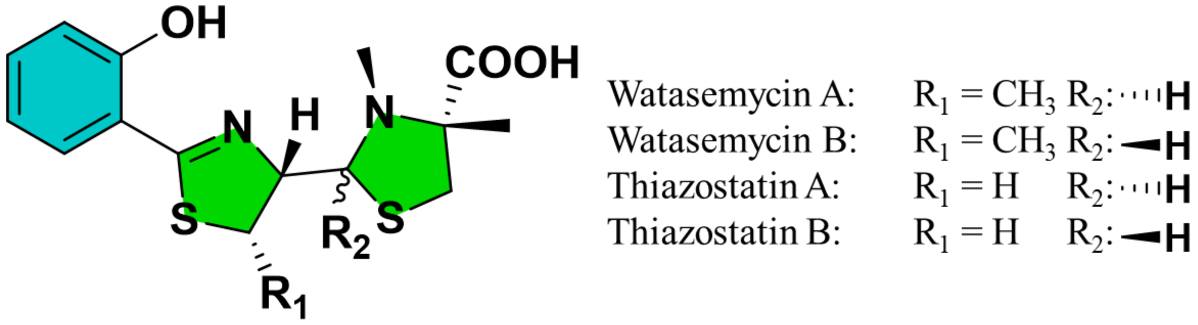

Figure S8. Structures of the previously reported compounds watasemycin A and B and thiazostatin A and B. The phenol and thiazole rings are highlighted in blue and green, respectively (Sasaki et al. 2002).

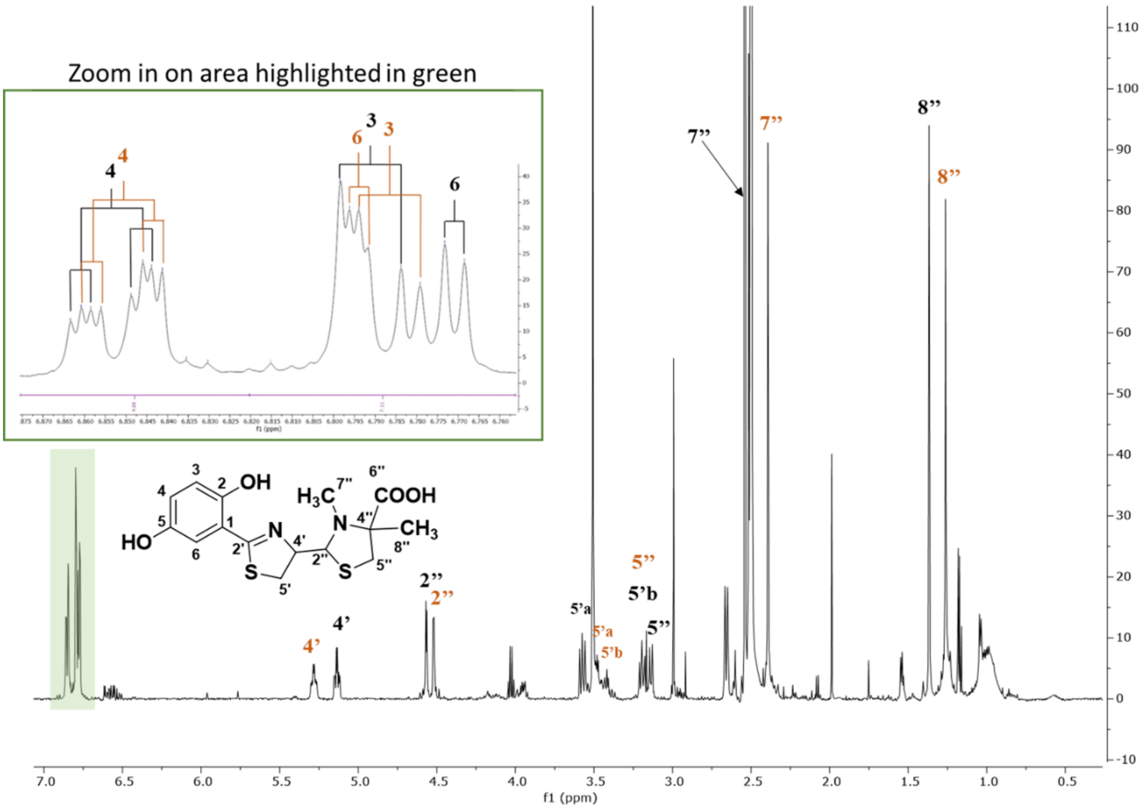

Figure S9. ^{1}H NMR (600 MHz, DMSO-d_6) spectrum of thiazostatin D (**7a**) and E (**7b**). Atoms from **7a** and **7b** are indicated with black and orange numbers, respectively.

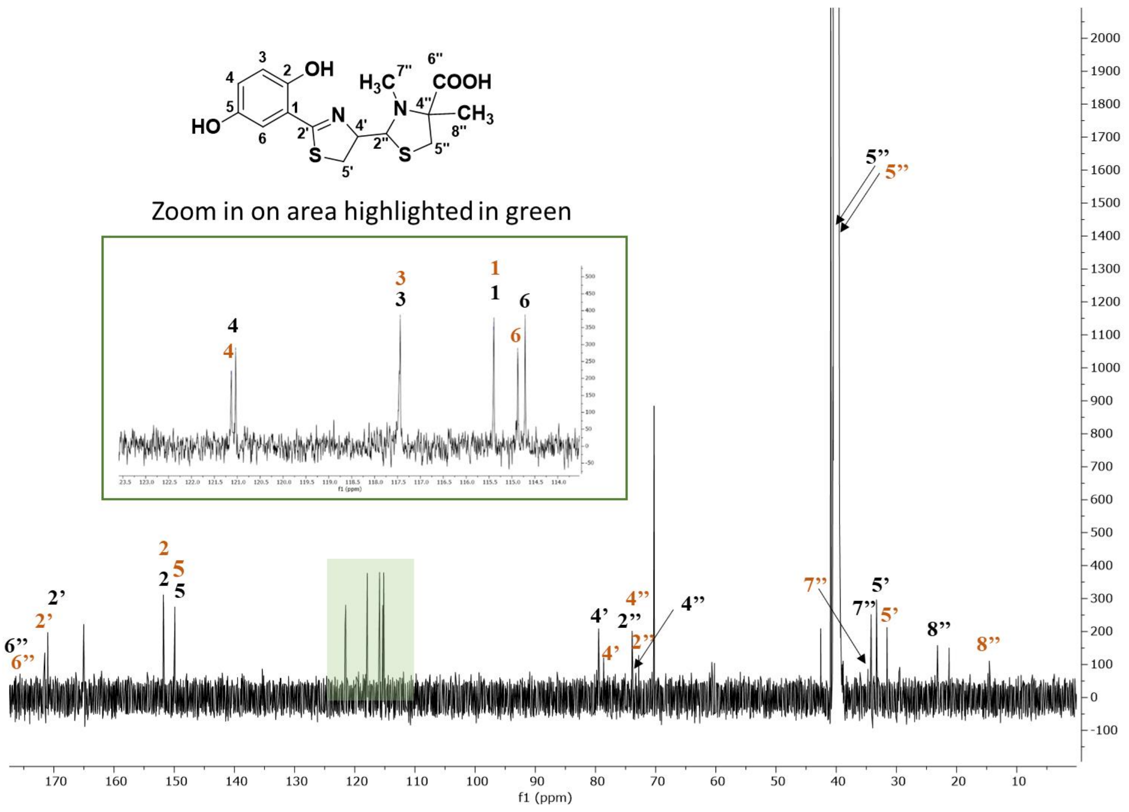

Figure S10. ^{1}C NMR (600 MHz, DMSO-d_6) spectrum of thiazostatin D (**7a**) and E (**7b**). Atoms from **7a** and **7b** are indicated with black and orange numbers, respectively.

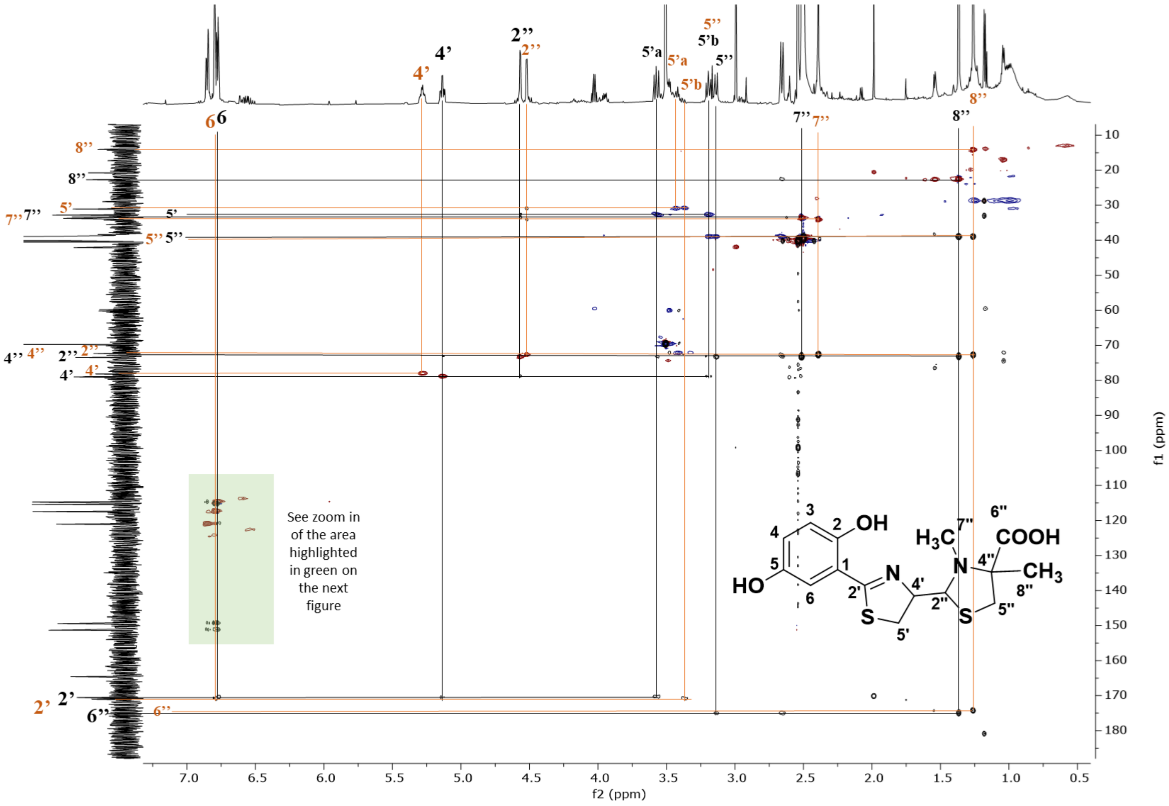

Figure S11. HSQC + HMBC (600 MHz, DMSO-d_6) spectrum of thiazostatin D (**7a**) and E (**7b**). Atoms from **7a** and **7b** are indicated with black and orange numbers, respectively.

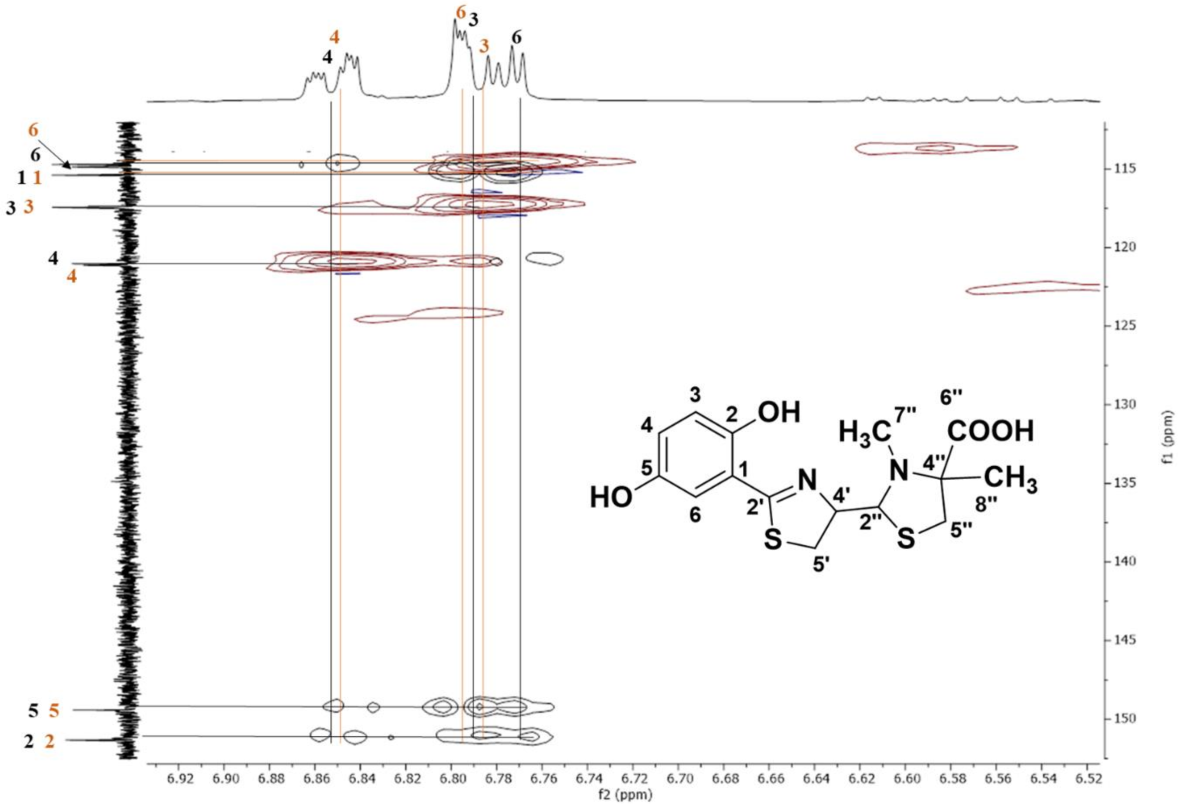

Figure S12. Zoom in on HSQC + HMBC (600 MHz, DMSO-d_6) spectrum of thiazostatin D (**7a**) and E (**7b**). Atoms from **7a** and **7b** are indicated with black and orange numbers, respectively.

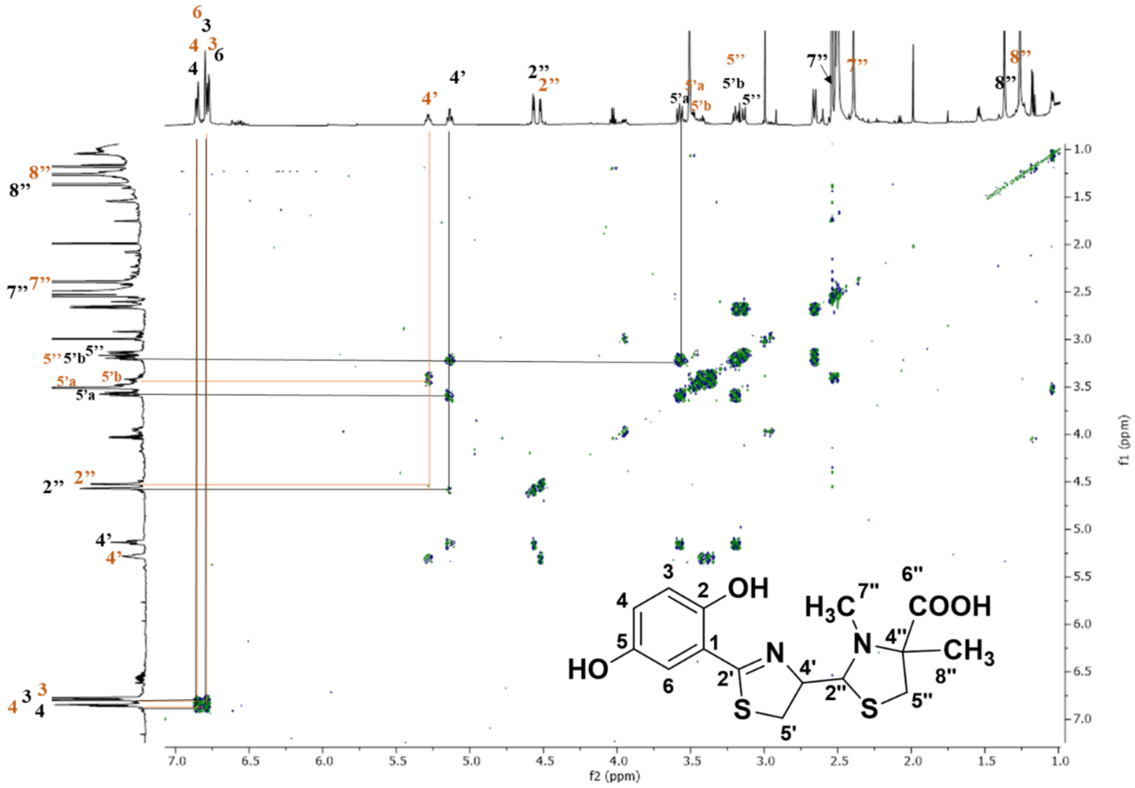

Figure S13. COSY (600 MHz, DMSO-d_6) spectrum of thiazostatin D (**7a**) and E (**7b**). Atoms from **7a** and **7b** are indicated with black and orange numbers, respectively.

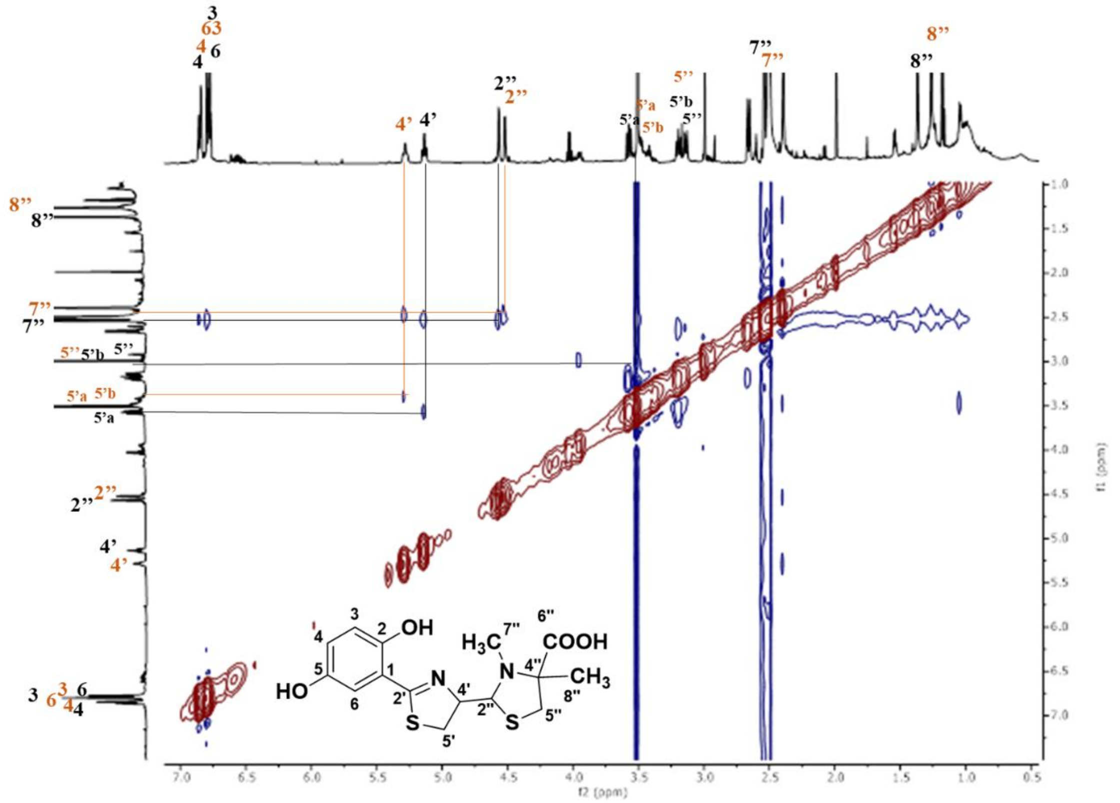

Figure S14. ROESY (600 MHz, DMSO-d_6) spectrum of thiazostatin D (**7a**) and E (**7b**). Atoms from **7a** and **7b** are indicated with black and orange numbers, respectively.

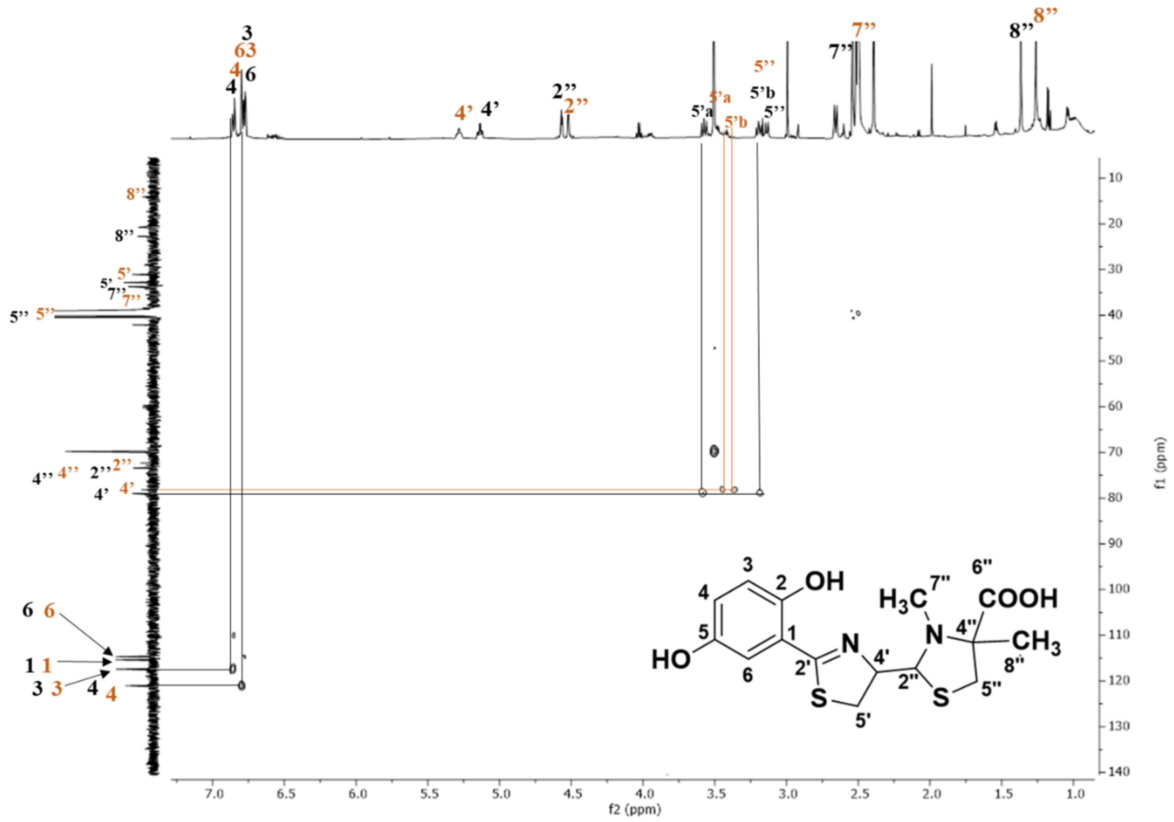

Figure S15. H2BC (600 MHz, DMSO-d_6) spectrum of thiazostatin D (**7a**) and E (**7b**). Atoms from **7a** and **7b** is indicated with black and orange numbers, respectively.

References

Danecek, Petr; Bonfield, James K.; Liddle, Jennifer; Marshall, John; Ohan, Valeriu; Pollard, Martin O. et al. (2021): Twelve years of SAMtools and BCFtools. In: *GigaScience* 10 (2). DOI: 10.1093/gigascience/giab008.

Li, Heng (2021): New strategies to improve minimap2 alignment accuracy. In: *Bioinformatics (Oxford, England)* 37 (23), S. 4572–4574. DOI: 10.1093/bioinformatics/btab705.

Sasaki, Omomitsu; Igarashi, Yasuhiro; Saito, Noriko; Furumai, Tamotsu (2002): Watasemycins A and B, new antibiotics produced by Streptomyces sp. TP-A0597. In: *J. Antibiot.* 55 (3), S. 249–255. DOI: 10.7164/ANTIBIOTICS.55.249.
